# Epigenetic regulation of diacylglycerol kinase alpha promotes radiation-induced fibrosis

**DOI:** 10.1038/ncomms10893

**Published:** 2016-03-11

**Authors:** Christoph Weigel, Marlon R. Veldwijk, Christopher C. Oakes, Petra Seibold, Alla Slynko, David B. Liesenfeld, Mariona Rabionet, Sabrina A. Hanke, Frederik Wenz, Elena Sperk, Axel Benner, Christoph Rösli, Roger Sandhoff, Yassen Assenov, Christoph Plass, Carsten Herskind, Jenny Chang-Claude, Peter Schmezer, Odilia Popanda

**Affiliations:** 1 Division of Epigenomics and Cancer Risk Factors, German Cancer Research Center (DKFZ), Im Neuenheimer Feld 280, 69120 Heidelberg, Germany; 2 Department of Radiation Oncology, Universitätsmedizin Mannheim, Medical Faculty Mannheim, University of Heidelberg, 68167 Mannheim, Germany; 3 Division of Cancer Epidemiology, German Cancer Research Center (DKFZ), Im Neuenheimer Feld 581, 69120 Heidelberg, Germany; 4 Division of Biostatistics, German Cancer Research Center (DKFZ), Im Neuenheimer Feld 581, 69120 Heidelberg, Germany; 5 Division of Preventive Oncology, German Cancer Research Center (DKFZ), Im Neuenheimer Feld 280, 69120 Heidelberg, Germany; 6 Lipid Pathobiochemistry Group, German Cancer Research Center (DKFZ), Im Neuenheimer Feld 280, 69120 Heidelberg, Germany; 7 Junior Research Group Biomarker Discovery, Division of Stem Cells and Cancer, German Cancer Research Center (DKFZ), Im Neuenheimer Feld 280, 69120 Heidelberg, Germany; 8 Biomarker Discovery, HI-STEM gGmbH, 69120 Heidelberg, Germany; 9 Instrumental Analytics and Bioanalytics, Technical University of Applied Sciences Mannheim, 68163 Mannheim, Germany; 10 German Center for Cardiovascular Research (DZHK), Partner Site Heidelberg/Mannheim, D-69120 Heidelberg, Germany

## Abstract

Radiotherapy is a fundamental part of cancer treatment but its use is limited by the onset of late adverse effects in the normal tissue, especially radiation-induced fibrosis. Since the molecular causes for fibrosis are largely unknown, we analyse if epigenetic regulation might explain inter-individual differences in fibrosis risk. DNA methylation profiling of dermal fibroblasts obtained from breast cancer patients prior to irradiation identifies differences associated with fibrosis. One region is characterized as a differentially methylated enhancer of diacylglycerol kinase alpha (*DGKA*). Decreased DNA methylation at this enhancer enables recruitment of the profibrotic transcription factor early growth response 1 (EGR1) and facilitates radiation-induced *DGKA* transcription in cells from patients later developing fibrosis. Conversely, inhibition of DGKA has pronounced effects on diacylglycerol-mediated lipid homeostasis and reduces profibrotic fibroblast activation. Collectively, DGKA is an epigenetically deregulated kinase involved in radiation response and may serve as a marker and therapeutic target for personalized radiotherapy.

## Abstract (web summary)

Radiotherapy can induce fibrosis in cancer patients, limiting its use in clinical settings. Here, the authors identify a differentially methylated enhancer of the lipid kinase DGKA in fibroblasts from breast cancer patients developing fibrosis after radiotherapy and they show that DGKA inhibition affects lipid homeostasis and reduces pro-fibrotic fibroblast activation.

## Graphical abstract

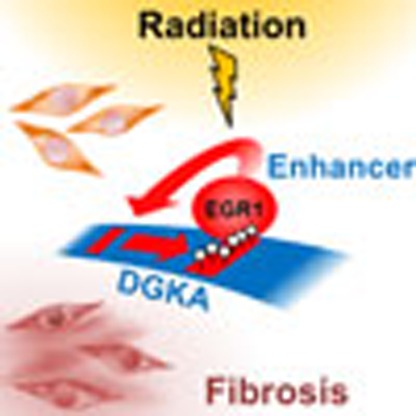

Radiation therapy is a common cancer treatment but the doses applied are often limited by the onset of adverse effects in the co-irradiated normal tissue. They can occur even months to years after radiotherapy and susceptibility differs widely among patients^1^. In breast cancer patients, fibrosis in the irradiated tissue is a frequent late reaction occurring in ∼20% of treated women^2^. Risk factors for radiation-induced fibrosis are not well-understood, but include genetic predisposition, mode of application and dose-related factors^1^. Radiation-induced fibrosis is characterized by increased connective tissue stiffness and loss of tissue function at the irradiated site. While its clinical features depend on the affected organ it was shown that common disease patterns exist on the molecular and cellular level^3^. Fibrotic tissue shows aberrant signalling by cytokines like transforming growth factor-beta 1 (TGFB1) and generation of permanently activated fibroblasts as key inducers of fibrogenesis^4^. Further fibrosis-associated signalling cascades include diacylglycerols (DAGs) that are known regulators of pleiotropic downstream signalling via activation of DAG-binding proteins such as protein kinase C (PKC)^5^,^6^. Cellular DAG levels are tightly regulated by ubiquitously expressed DAG kinases that limit DAG production via metabolism of DAG to phosphatidic acid (PA)^7^,^8^. However, the role of DAG kinases in fibrotic processes has remained unexplored.

Given the irreversible nature of fibrotic disease much effort has been made to identify risk factors for radiation fibrosis to adjust radiotherapy to individual patient susceptibility. Genetic variants defined by single nucleotide polymorphisms have been studied as predictors for radiation toxicity^9^, but these markers cannot fully explain the high incidence of radiation-induced fibrosis^10^. Epigenetic regulation has emerged as a potential mechanism of various diseases including fibrosis^11^,^12^,^13^. The field of epigenetics comprises pathways and regulatory features that control genomic activity without changes in the DNA sequence. Epigenetic modifications include DNA methylation, histone modifications, non-coding RNAs and three-dimensional chromatin organization^14^. Recent findings revealed the establishment of aberrant DNA methylation patterns in fibrosis^12^. Importantly, epigenetic differences may already be present before disease onset and could therefore be used as predictive markers for risk estimation. It has been shown that epigenetic changes are associated with fibrosis susceptibility *in vivo*^15^. The links between epigenetic alterations and downstream events in fibrosis remain, however, often unknown. Comprehensive genome-wide DNA methylation analyses of epigenetic patterns^14^ in radiation-induced fibrosis have not been done. Therefore, we based the present study on the hypothesis that pre-existing DNA methylation differences in unirradiated primary human fibroblasts influence the risk of later fibrosis development after radiotherapy. Here we identified DGKA as an epigenetically deregulated kinase involved in fibroblast activation after irradiation and stress exposure which has potential to serve as a therapeutic target for preventing radiation-induced fibrosis.

## Results

### Genome-wide DNA methylation and radiation-induced fibrosis

**Figure 1:**
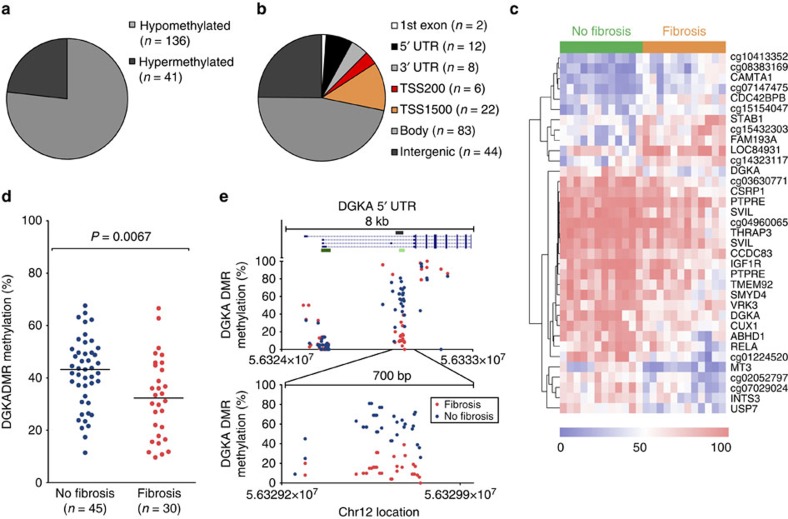
Differential DNA methylation of *DGKA* and fibrosis. Distribution of differential methylation (**a**) and genomic site distribution (**b**) in a representative set of differentially methylated CpG sites (*P* value <0.05 after beta regression; *n*=177) from Illumina 450 K array data according to the adjusted *P* values. (**c**) A heat map of Illumina 450 K DNA methylation values at differentially methylated sites (*n*=35, *P*<0.05 (adjusted Wald test) estimated methylation difference >10%) separating fibrosis (orange) and non-fibrosis (green) samples. Rows show gene names or CpG site IDs when gene annotation is not available. Scale bar indicates DNA methylation (in %). (**d**) Differential methylation at the *DGKA* locus measured by EpiTYPER technology in a total sample set of 75 patient-derived fibroblasts. *P* value was determined using Wilcoxon rank-sum testing. Bars indicate median. (**e**) Detailed interrogation of DNA methylation using EpiTYPER in two distinct patient fibroblasts (fibrosis and fibrosis-free) at the *DGKA* 5′ UTR (upper panel) and the differentially methylated region (lower panel). Each dot represents an individual CpG per patient from EpiTYPER analysis. The genomic DMR region interrogated (grey), CpG islands (green) and *DGKA* transcripts (blue) are shown. Intergenic, no gene context assigned; body, gene body/intragenic localization; TSS200/1,500: 200 bp/1,500 bp window around transcription start site; UTR, untranslated region; 1st exon, localization within the first exon of a known transcript.

To identify sites of differential DNA methylation associated with radiation-induced fibrosis risk, we screened 24 primary human dermal fibroblast samples derived from breast cancer patients receiving intraoperative radiotherapy (IORT; Supplementary Table 1). Samples consisted of two groups: cells derived from patients either (i) developing radiation-induced fibrosis at the irradiated breast within a follow-up time of 2–5 years after IORT (*n*=12) or (ii) currently fibrosis-free (*n*=12). Genome-wide DNA methylation levels of >450,000 single CpG sites were measured using Infinium HumanMethylation450 BeadChips. Using the non-fibrosis group as a reference, beta regression analysis with adjustment for patients' age, smoking status and exposure to chemotherapy was performed to obtain a list of differentially methylated CpGs. Differential methylation was observed for 177 CpG sites with an adjusted *P* value <0.05 (adjusted Wald test), most frequently exhibiting a loss of DNA methylation (hypomethylation) (Fig. 1a) and at non-promoter sites (Fig. 1b). Further filtering of differentially methylated CpGs by including only sites showing ≥10% methylation difference or gene loci with ≥2 differentially methylated CpG sites identified 35 differentially methylated candidate sites that distinguished the fibrosis and non-fibrosis groups (Fig. 1c, Supplementary Table 2). The DAG kinase alpha (*DGKA*) locus was of particular interest as it contained two differentially methylated CpG sites in a single intragenic region and showed one of the strongest methylation differences. We confirmed the fibrosis-associated *DGKA* hypomethylation using EpiTYPER technology as an independent quantitative method (Supplementary Fig. 1a–c) in an expanded sample set of overall 75 patients (Fig. 1d, Supplementary Table 1). Furthermore, a detailed comparison between a lowly methylated sample associated with fibrosis onset and a highly methylated sample from the control group indicated a pronounced and spatially well-defined loss of DNA methylation within an intragenic CpG island in the fibrosis sample (Fig. 1e). This differentially methylated region (DMR) was highly methylated in DNA of patient-derived blood cells irrespectively of the fibrosis status, thus showing the cell-type specificity of differential methylation (Supplementary Fig. 1d).

### Epigenetic state of *DGKA* linked to inducible transcription

**Figure 2:**
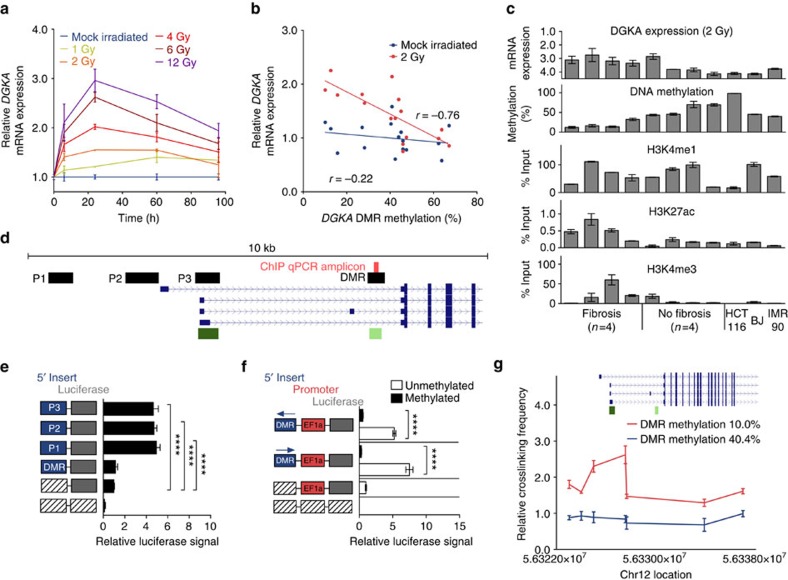
The *DGKA* DMR shows gene enhancer characteristics. (**a**) *DGKA* mRNA expression in primary human dermal fibroblasts on exposure to increasing doses of gamma irradiation. Data depict mean±s.e.m. from duplicate experiments in three different normal human dermal fibroblast strains derived from healthy donors. (**b**) Radiation-inducible and constitutive *DGKA* mRNA expression correlated with DNA methylation at the intragenic *DGKA* DMR in dermal fibroblasts (*n*=16). (**c**) Chromatin immunoprecipitation in differentially methylated patient-derived fibroblasts (*n*=8) and hypermethylated control cell lines (BJ, IMR90, HCT116) for enhancer histone marks (H3K4me1 and H3K27ac), as well as promoter marks (H3K4me3) at the *DGKA* DMR. Data depict mean±s.e.m. of four independent immunoprecipitations (triplicates for mRNA and DNA methylation measurements) per fibroblast strain. (**d**) Map of the interrogated DGKA 5′ UTR with CpG islands (green), *DGKA* transcripts (blue), ChIP-quantitative PCR amplicons (red) and luciferase reporter inserts (black). (**e**) Luciferase assay in pGL 4.10-based reporters measuring promoter activity in three different upstream elements P1, P2 and P3 (for their location, see **d**) and the *DGKA* DMR region. (**f**) Influence of sequence orientation (arrow to the left/right: sense/antisense orientation) and DNA CpG methylation (grey/black bars: unmethylated/methylated insert) on the *DGKA* DMR gene enhancer activity in a luciferase reporter plasmid carrying a CpG-free EF1alpha minimal promoter (EF1a). Graphs (**e**,**f**) depict mean±s.e.m. of four independent replicates in HEK293T cells. Vector maps (**e**,**f**) indicate DMR inserts (blue), minimal promoter (red) and luciferase (grey), hatched boxes indicate lack of vector insert. (**g**) Chromatin conformation capture depicting the interaction of the *DGKA* DMR with the surrounding *DGKA* locus in two patient fibroblasts with low- or high-DMR methylation. Data show mean±s.e.m. of three independent experiments. Insert shows gene regions tested with DGKA transcripts depicted in blue and CpG islands in green. ****P<0.0001, Student's *t*-test. DMR, differentially methylated region; UTR, untranslated region.

Due to the known involvement of DAGs and associated signalling lipids in fibrotic processes^16^, we investigated the epigenetic regulation of *DGKA* to understand the potential regulatory role of DNA methylation in radiation-induced fibrosis. In dermal fibroblasts, *DGKA* mRNA expression increased 24 h after ionizing radiation in a dose-dependent manner and was still detectable after 96 h (Fig. 2a). Applied doses resembled single dose fractions used in conventional radiotherapy (2 Gy fractions)^2^ and healthy tissue exposure in IORT treatment regimens^17^,^18^. Increased transcription after radiation was inversely correlated (*r*=−0.76, *P*=0.0007, F test) with the presence of DNA methylation at the *DGKA* DMR (slope=−0.021, 95% confidence interval (CI)=−0.031,−0.011), whereas *DGKA* expression in unirradiated cells was not correlated (*r*=−0.22, *P* value=0.43, F test) with the methylation status of this region (slope=0.004; CI=−0.013-0.006). These data suggest that the DMR influences the induction of *DGKA* by radiation (Fig. 2b, Supplementary Fig. 2a). We compared radiation-induced upregulation of the DGKA protein in fibroblasts from patients developing fibrosis with low methylation (*n*=4) and in controls with high methylation (*n*=4) by western blot (Supplementary Figs 3 and 4), DAG kinase activity assay (Supplementary Fig. 2c) and quantitative protein mass spectrometric analysis (Supplementary Fig. 2b) confirming methylation-dependent DGKA induction and increased activity after radiation. It has been shown that gene regulatory regions can function as enhancers and thereby modulate gene expression. Such regions are characterized by specific patterns of enhancer-associated histone modifications^19^ like histone H3 lysine 27 acetylation (H3K27ac) and lysine 4 mono- and trimethylation (H3K4me1, H3K4me3), while gene promoters are characterized by an excess of H3K4me3 over H3K4me1. We therefore investigated these histone marks at the *DGKA* DMR using chromatin immunoprecipitation (ChIP) and found gene enhancer characteristics (Fig. 2c, Supplementary Fig. 5). The H3K27ac mark was mainly present in fibroblasts with low methylation at the DMR, suggesting an inverse association of this activating histone mark with DNA methylation levels. For further confirmation, we compared the presence of H3K27ac with DGKA amount and activity and found a positive correlation with radiation-induced DGKA levels (Supplementary Fig. 6c,d).

The identified DNA methylation and histone marks at the *DGKA* DMR were not found to be significantly altered after exposure to radiation (72 h) or to the activating cytokine TGFB1 (120 h). This was shown in fibroblasts from five patients with a wide range of *DGKA* DMR methylation (Supplementary Fig. 7a–c). In addition, DNA methyltransferase (DNMT) expression and activity did not show significant differences in patient fibroblasts (Supplementary Fig. 7d,e). Furthermore, prolonged *in vitro* cultivation of normal human dermal fibroblasts (NHDFs) did not significantly alter *DGKA* expression or its methylation (Supplementary Fig. 7f,g). Taken together, these results indicate a stable epigenetic pattern at the *DGKA* DMR. We therefore conclude that this DMR modulates radiation-induced *DGKA* transcription either as an alternative promoter site or as a gene enhancer. We tested both possibilities in luciferase reporter assays interrogating the ability of the *DGKA* DMR region to induce luciferase gene expression when inserted as a promoter (Fig. 2e) or as an upstream gene enhancer (Fig. 2f). Promoter activity was identified for three upstream regions P1, P2 and P3 (Fig. 2d, Supplementary Fig. 8) but not for the DMR (Fig. 2e). When combined with a minimal promoter, the *DGKA* DMR showed strongly increased luciferase signals in an orientation-independent manner indicative of enhancer activity. *In vitro* methylation of the reporter construct abrogated the enhancer function and confirmed the role of DNA methylation in the regulatory activity of the DMR (Fig. 2f). Chromatin conformation capture (3C) at the *DGKA* 5′ untranslated region showed that the *DGKA* DMR interacts with the upstream DGKA promoter specifically in a fibroblast sample with low-DMR methylation (Fig. 2g). In summary, our data indicate that the *DGKA* DMR is a radiation-inducible gene enhancer in dermal fibroblasts.

### Transcriptional induction of *DGKA* involves binding of EGR1

**Figure 3:**
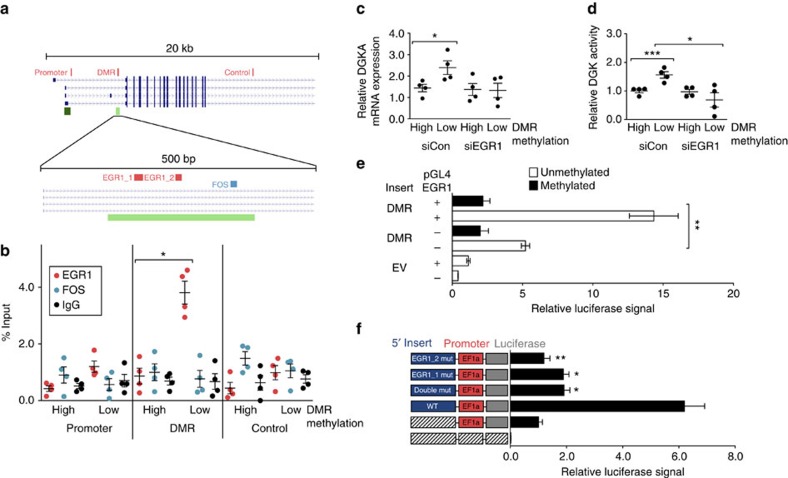
The *DGKA* DMR is regulating DGKA activation in fibroblasts. (**a**) Upper panel: map of the interrogated region at the *DGKA* gene (transcripts shown in blue), CpG islands (green) and location of PCR amplicons used in ChIP analysis (red). lower panel: putative transcription factor-binding sites for EGR1 and FOS identified by *in silico* prediction at the *DGKA* DMR. (**b**) ChIP analysis showing EGR1 and FOS signals at the *DGKA* locus in patient fibroblasts with differential *DGKA* DMR methylation (high methylation, *n*=4; low methylation, *n*=4). Dots show mean values from four independent immunoprecipitations per fibroblast sample, bars depict mean±s.e.m. in each group. Rabbit IgG served as control. (**c**,**d**) *DGKA* mRNA transcription 48 h after siRNA-mediated knockdown of EGR1 in irradiated (2 Gy) patient-derived fibroblasts (*n*=4 high-methylation and *n*=4 low-methylation fibroblasts) (**c**) and global DGK activity (**d**). siCon, non-targeting siRNA control, siEGR1, siRNA directed against *EGR1.* Dots show mean values from duplicate experiments for each fibroblast (triplicates for **d**), bars depict mean±s.e.m. for each group. (**e**) Luciferase reporter assay in HEK293T cells on overexpression of EGR1 showing signal from a reporter containing the *DGKA* DMR and an adjacent minimal promoter (EF1a). Grey/black bars: unmethylated/methylated insert after *in vitro* methylation of the reporter plasmid. (**f**) Site-directed mutagenesis of the two predicted EGR1-binding sites at the *DGKA* DMR and influence on enhancer activity in a luciferase reporter construct. Relative luciferase activity in a reporter vector carrying the *DGKA* DMR (blue), a minimal promoter (EF1a, red) and luciferase (grey) are shown. *P* values refer to comparison with WT-EF1a reporter. Data from **e** and **f** show mean±s.e.m. of at least four individual measurements. EF1a, CpG-free EF1alpha minimal promoter; EV, empty vector; WT, wild type; hatched boxes indicate lack of vector insert *P<0.05, **P<0.01, ***P<0.001, Student's *t*-test.

The identification of enhancer activity at the *DGKA* DMR, as well as previous reports of transcription factor binding from the ENCODE project data^20^ (Supplementary Fig. 9a) prompted us to analyse the regulation of radiation-inducible *DGKA* expression. *In silico* prediction of transcription factor-binding motifs at the *DGKA* DMR (Fig. 3a,b; Supplementary Fig. 9b; Supplementary Table 3) revealed binding sites of several factors including FBJ murine osteosarcoma viral oncogene homologue (FOS) and early growth response 1 (EGR1). The latter two factors were further investigated as both have been implicated in fibrosis^21^,^22^ and radiation response^23^,^24^. Our ChIP analyses in patient fibroblasts did not reveal FOS binding in untreated (Fig. 3b) or stress-exposed fibroblasts (Supplementary Fig. 10a–d), while genes previously reported as FOS- and EGR1-binding sites showed corresponding ChIP signals (Supplementary Fig. 10e). EGR1 binding was identified specifically at the *DGKA* DMR and only in fibroblasts with low-DMR methylation (Fig. 3b), indicating that this region is a functional EGR1-binding site in fibroblasts with low DNA methylation at this region. High-resolution DNA methylation data at the DMR region showed that the conserved 5′ EGR1-binding motif (EGR1_1) contains two CpG sites and that these sites were indeed differentially methylated in patient fibroblasts (Supplementary Fig. 11). This suggested a direct modulation of EGR1 binding by DNA methylation. To link EGR1 to *DGKA* induction after radiation, we tested the effect of siRNA-mediated EGR1 knockdown on *DGKA* mRNA expression and DAG kinase enzymatic activity in patient fibroblasts (*n*=8) after radiation. Irradiated fibroblasts with high-*DGKA* DMR methylation showed no change in *DGKA* inducibility after EGR1 knockdown. Fibroblasts with low-DMR methylation revealed a significant increase in radiation-induced *DGKA* mRNA expression (Fig. 3c) and enzymatic activity (Fig. 3d). This increase was no longer observed after EGR1 silencing. We therefore conclude that induction of *DGKA* is mediated by EGR1 selectively in fibroblasts having low methylation at the *DGKA* DMR and thus at the previously defined EGR1-binding site. In addition, we measured the effect of EGR1 overexpression and DNA methylation on the DMR enhancer activity using luciferase reporter assays. We found that increased EGR1 drives luciferase activity mediated by the DMR. This effect was inhibited by DNA methylation (Fig. 3e). We further compared the enhancer activity of the *DGKA* DMR in luciferase reporters containing either a wild-type or a mutated DMR lacking two consensus EGR1-binding sites. We observed that mutation of the EGR1-binding motifs significantly impaired enhancer activity (Fig. 3f). Taken together, our data reveal a previously undescribed role of the *DGKA* DMR as an EGR1-binding gene enhancer element controlling *DGKA* transcription after radiation exposure.

### DGKA regulates profibrotic activation and lipid signalling

**Figure 4:**
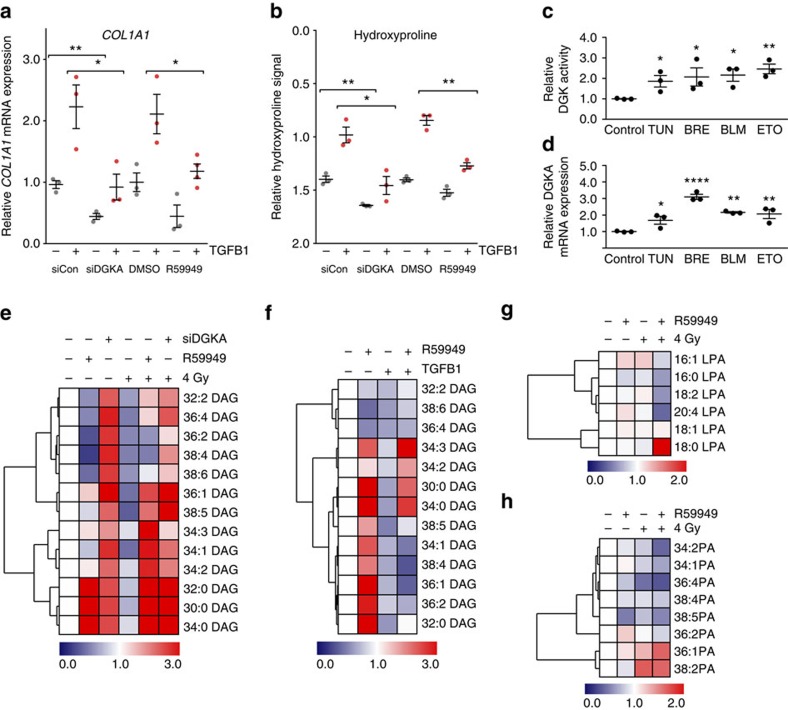
A role of *DGKA* in fibroblast activation and stress response. (**a**,**b**) Collagen expression in untreated and TGF-beta1-stimulated NHDF (*n*=3) after siRNA silencing or inhibition of DGKA using the small molecule inhibitor R59949 (5.0 μM). Cells were pre-treated with drug or siRNA for 48 h, subsequently treated with TGFB1 (4 ng ml^−1^) and analysed 48 h later. Graphs show expression of collagen 1A1 *COL1A1* mRNA (**a**) and total collagen release using hydroxyproline content as a surrogate marker (**b**). (**c**) Diacylglycerol kinase (DGK) activity and (**d**) DGKA mRNA expression in NHDF (*n*=3) on treatment for 48 h with model stress inducers, BLM, 40 μM bleomycin; BRE, 0.1 μM brefeldin A; ETO, 30 μM etoposide; TUN, 0.5 μM tunicamycin; *P* values refer to comparison with untreated controls. Dots show mean values from duplicate experiments for each fibroblast (triplicates for **d**), bars depict mean±s.e.m. in each group. (**e**) DAG levels in NHDF after inhibition of DGKA by R59949 (5.0 μM) or siRNA 48 h after co-exposure to radiation. (**f**) DGKA inhibition by R59949 and changes in DAG levels in NHDF co-exposed to TGFB1 (4 ng ml^−1^) for 48 h. (**g**,**h**) LPA (**g**) and PA (**h**) levels in NHDF after inhibition of DGKA by R59949 (5.0 μM) and co-exposure to radiation. Scale bars indicate normalized relative DAG or PA/LPA levels in fibroblasts compared with untreated controls (decrease, blue; increase, red). Data depict mean from duplicate experiments in NHDF (*n*=3). siCon, non-targeting siRNA control; siDGKA, siRNA directed against *DGKA*. *P<0.05, **P<0.01, ****P<0.0001, Student's *t*-test.

We further hypothesized that DGKA plays a role in fibroblast stress response linking aberrant epigenetic regulation of *DGKA* to radiation-induced fibrosis. To test this hypothesis, we used a cellular model of NHDF activation by TGFB1 as a well-established profibrotic stimulus^4^. TGFB1 stimulation increased two markers of fibroblast activation: collagen 1A1 (*COL1A1*) mRNA expression (Fig. 4a) and total secreted hydroxyproline, a marker representative for total collagen deposition (Fig. 4b). *DGKA* downregulation via siRNA or its specific inhibition by R59949 (ref. 25) significantly reduced the TGFB1 response of both markers. In a similar approach, we used ionizing radiation and the radiomimetic drug bleomycin for fibroblast activation. We again found a significant repression of fibroblast activation markers such as *COL1A1* and alpha smooth muscle actin (*ACTA2*; Supplementary Fig. 12a). Further stress mimetic compounds including the DNA damaging agent etoposide and the endoplasmic reticulum (ER) stress mimetics tunicamycin and brefeldin A, also increased global DAG kinase activity (Fig. 4c) and *DGKA* mRNA expression (Fig. 4d) in NHDFs. The most pronounced DGKA induction was seen after DNA damaging or ER stress-inducing treatments, but not after oxidative stress inducers (Supplementary Fig. 12b).

In T cells, DGKA was shown to exert its downstream effects via modulation of DAG turnover^7^. DAGs are phosphorylated by DGKA to limit DAG signals^8^. We therefore investigated the effect of DGKA inhibition on 13 DAGs (Supplementary Table 5) previously reported to be altered in DGKA knockout mice^26^. Using siRNA or R59949 in NHDFs, we revealed an increase of 7 out of 13 analysed DAGs on DGKA inhibition, with saturated DAGs (30:0 DAG, 32:0 DAG and 34:0 DAG) showing the most pronounced effects (Fig. 4e). NHDFs displayed *DGKA* induction after radiation (Fig. 2a) which coincided with decreases in DAGs. These observations suggested a key role for DGKA in stress-induced events via DAG signalling. We further showed that TGFB1-mediated fibroblast activation was associated with reduction of DAGs. This effect was partially mitigated by co-treatment with R59949 (Fig. 4f). Downstream effects of alterations to DAG pools involve processing of PAs to lysophosphatidic acid (LPA)^27^, a key profibrotic mediator^28^,^29^,^30^. We therefore investigated whether DGKA modulation also affects PA and LPA levels. We found that inhibition of DGKA by R59949 reduced specific PAs and LPAs in NHDF and these effects were even stronger on radiation exposure (Fig. 4g,h, Supplementary Table 6). *In vitro* exposure of NHDF to LPA induced collagen, but also *DGKA* mRNA expression. Both effects were attenuated by inhibition of DGKA with R59949 (Supplementary Fig. 13a–d). Inhibition was more pronounced after LPA exposure than after TGFB1 stimulation. Similarly, radiation-induced collagen and *DGKA* expression were weakened by the LPA receptor antagonist Ki16425 (Supplementary Fig. 13e,f), indicating an interconnection of radiation response, LPA and DGKA signalling. These results underline the importance of DGKA in both stress response and profibrotic signalling. Downstream effects mediated by radiation-induced DGKA activity were linked to altered DAG-mediated lipid signalling in NHDF.

### DGKA in stress signalling networks in patient fibroblasts

**Figure 5:**
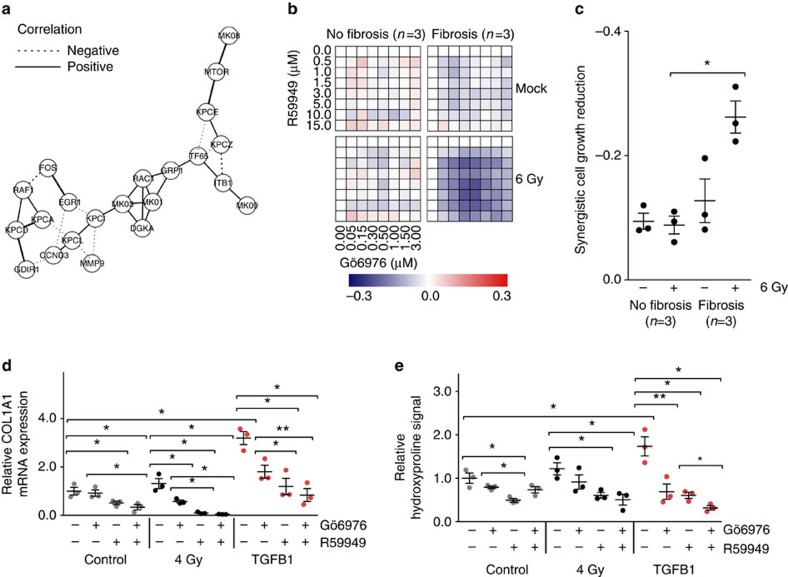
Co-inhibition of DGKA and PRKCA in irradiated fibroblasts. (**a**) Partial correlation network of DGKA-associated proteins in fibroblasts. Data are based on correlations of relative protein signals (unirradiated versus irradiated) in seven patient fibroblasts. Line thickness indicates the strength of correlation, dotted lines depict inverse correlation (**b**) Effect of inhibitors of DGKA (R59949) and PRKCA (Gö6976) on cell growth of fibroblasts from fibrosis or non-fibrosis patients exposed to ionizing radiation at various dose combinations. Data show cell growth determined in quadruplicates after 96h drug treatment with radiation applied after 48 h. Cell growth was measured by calcein fluorescence assay. (**c**) Additive or synergistic drug effects from **b** were calculated with the Bliss independence model comparing the expected (additive) effects with the observed effects. Scale bar depicts the observed viability differences in comparison to the expected additive effects as blue (synergistic growth suppression) to red (synergistic growth increase). (**d**,**e**) Impact of R59949 (5.0 μM) and Gö6976 (0.5 μM) on collagen synthesis in NHDF measured by *COL1A1* mRNA expression (**d**) or hydroxyproline release (**e**). Cells were pre-treated with drugs for 48 h and analysed an additional 48 h after exposure to radiation or TGFB1 (4 ng ml^−1^) with concurrent drug treatment. Dots show mean values from duplicate (**d**,**e**) or quadruplicate (**c**) experiments for each fibroblast, bars depict mean±s.e.m. in each group. *P<0.05, **P<0.01, Student's *t*-test.

Our results indicated a potential role of altered DGKA expression in profibrotic stress signalling. This prompted us to search the literature for proteins associated with DGKA function. The search resulted in 20 candidate proteins (Supplementary Table 7), which were reported to be expressed in various tissues but no data on DGKA signalling in fibroblasts were available. It has been reported that functional protein associations and co-regulation can be inferred by partial correlation networks of mRNA or protein expression data^31^. We therefore investigated the expression of candidate proteins as well as EGR1 and DGKA by quantitative mass spectrometry in patient-derived fibroblasts (*n*=7) with various *DGKA* DMR methylation levels either untreated or 48 h after irradiation. To identify proteins co-regulated on radiation exposure, we estimated sample partial correlation coefficients for DGKA and the selected candidates, tested for significance of these coefficients and created a partial correlation network. Correlations (*P* value for partial correlation <0.1) were observed between DGKA and the mitogen-activated protein kinases 1 and 3 (MAPK1, *P*=0.039 and MAPK3, *P*=0.047, Bayes Analysis^32^; Supplementary Table 7) and Ras-related C3 botulinum toxin substrate 1 (RAC1, *P*=0.06, Bayes Analysis^32^) (Fig. 5a).

When considering all correlations among the 22 proteins, most interconnections with DGKA were indirect and linked through other proteins of the network. PKC isoforms represented distinct nodes with potential impact on DGKA signalling (Fig. 5a). PKC alpha (PRKCA, KPCA in Fig. 5a) was selected for further investigation because of pronounced expression in fibroblasts (Supplementary Fig. 14) and previous reports about its role in profibrotic signalling^33^. Protein and mRNA expression data indicated increased expression of PRKCA (Supplementary Figs 15 and 16) in fibroblasts of patients developing radiation fibrosis. To characterize the interaction of both signalling kinases, we measured cell proliferation as a fibroblast activation marker and tested for combined effects of R59949 and the PRKCA inhibitor Gö6976 (ref. 34). Effects of both drugs applied either separately or in combination were measured. Two groups of fibroblasts were used, either from patients developing or not developing fibrosis, with strong and weak *DGKA* inducibility, respectively (Fig. 5b). Applying the Bliss independence model^35^, additive reduction of cell growth after combined treatment was observed in all fibroblasts tested. However, the additional induction of *DGKA* with ionizing radiation selectively led to synergistic growth impairment in fibroblasts from patients later developing fibrosis (Fig. 5c) that were characterized by lower *DGKA* DMR methylation and increased DGKA inducibility (Supplementary Fig. 17). This synergism was recapitulated in experiments with the *DGKA*- and *EGR1*-inducing stress stimuli bleomycin, etoposide, brefeldin and tunicamycin using calcein fluorescence and bromodeoxyuridine (BrdU) incorporation assays (Supplementary Fig. 18a–e). Further fibroblast activation markers, such as collagen synthesis (*COL1A1*, *COL1A2* and *COL3A1* mRNA expression) and total hydroxyproline release, were investigated after treatment with the two inhibitors using concentrations that as single treatments did not impair cell growth by >20% (5.0 μM R59949 and 0.5 μM Gö6976; Supplementary Fig. 19). We observed a reduction of all markers after DGKA or PRKCA inhibition. With combined treatment, this reduction was significantly stronger (Fig. 5c,d, Supplementary Figs 20 and 21). In summary, we found synergistic effects on cell growth and fibroblast activation after inhibition of DGKA and PRKCA in fibroblasts from patients developing fibrosis. Importantly, these effects were modulated by differential methylation of the intragenic enhancer region in *DGKA* that determined DGKA protein levels in fibroblasts, particularly the inducibility after radiation and DGKA-mediated cellular stress response (Supplementary Fig. 22). Thus, altered *DGKA* DMR methylation shows a functional impact on DGKA signalling and associated proteins.

## Discussion

We analysed fibroblasts isolated prior to treatment of breast cancer patients undergoing radiotherapy and identified DNA methylation differences in unirradiated fibroblasts, which were associated with fibrosis development after radiation. We focused our investigations on a DMR at the *DGKA* locus that was less methylated in patients developing fibrosis compared with those that did not. Confirmation of this association in an expanded set of 75 patient samples suggests that the *DGKA* DMR is a novel marker for fibrosis risk. This finding contributes to the growing evidence of altered DNA methylation in the context of fibrotic diseases, including radiation fibrosis^11^,^36^. DGKA is known to phosphorylate the lipid messenger DAG and controls DAG-mediated signalling relevant for fibrosis^7^,^8^. To substantiate the differential *DGKA* methylation with mechanistic evidence, we investigated whether DGKA is functionally related to both radiation response and fibroblast activation. *In vitro* irradiation increased *DGKA* mRNA and protein levels in fibroblasts with low-*DGKA* methylation. Radiation inducibility has already been reported for *DGKA* in expression profiling experiments^37^. Here we show that *DGKA* induction after radiation is highly variable between individual patients and is epigenetically regulated through an intragenic stress-inducible enhancer. Our work revealed that the *DGKA* DMR serves as a binding site for the profibrotic key regulator EGR1 (ref. 38). This observation is in line with the reported involvement of *EGR1* in cellular stress response, with the kinetics of *DGKA* induction following that of *EGR1* (ref. 23). Given the widespread involvement of EGR1 in fibrotic diseases^22^,^33^,^39^, we suggest a more common function of DGKA and its epigenetic regulation in fibrosis. Indeed, variable *DGKA* DNA methylation does not seem to be restricted to breast cancer patients as it was also found in fibroblasts of a previously published cohort of healthy donors although a potential association with fibrotic disease was not evaluated^40^.

Studies in T cells have revealed that DGKA inhibits MAPK signalling via reduction of DAG and functional inhibition of the downstream DAG-binding protein RASGRP1 (refs 7, 8). In fibroblasts, RASGRP1 protein levels were low and showed low to absent signals in RNA sequencing analysis. We therefore propose that DGKA is implicated in alternative signalling pathways in fibroblasts. Indeed, DGKA levels were highly correlated with kinases of the MAPK pathway. In line with our data, DGKA was shown to activate cell proliferation and MAPK1/3 signalling during liver carcinogenesis^41^, a process tightly linked to fibrotic tissue remodelling and epigenetic aberrations^42^,^43^. MAPK signalling was also reported to activate EGR1 in human lung epithelial cells exposed to silica as a profibrotic stimulus^39^ and in kidney fibrosis^33^. In our fibroblasts, EGR1 was involved in radiation-related *DGKA* induction by binding to the unmethylated DMR. Thus, a positive feedback loop might exist that is mediated by MAPK signalling and driven by epigenetic deregulation at the *DGKA* DMR. If such a mechanism escapes signalling control it may result in persisting fibroblast activation and subsequent fibrosis development. EGR1 has been reported to be a direct mediator of profibrotic events such as collagen gene induction by TGFB1 (ref. 44). However, EGR1 responses are often rapid and transient^23^,^44^, which stand in sharp contrast to the chronic and progressive nature of fibrosis. We therefore hypothesize that the profibrotic long-term effects of EGR1 in fibroblasts may be driven by the induction of profibrotic signalling networks that include DGKA and could prolong EGR1 action at its genomic targets.

We observed a strong impact of DGKA inhibition and radiation on cellular levels of several DAGs, PAs and LPAs. We propose that the observed decline of DAG levels after irradiation is mediated by DGKA induction and subsequent phosphorylation of DAGs. In line with previous reports^26^, impairment of DGKA resulted in increases of various DAG molecules, as well as decreases in LPAs. Strikingly, the effects we observed were heterogeneous among individual DAG and PA/LPA subclasses, with saturated side chain lipids being more strongly affected. Global deregulation of saturated lipid processing was shown to be profibrotic^45^. We have also found that saturated side chain DAGs were strongly affected by TGFB1 and co-treatment with a DGKA inhibitor. Specific PAs and LPAs also showed pronounced changes on DGKA inhibition. These subclasses of lipids have thus been identified as potential molecular mediators of DGKA downstream signalling after profibrotic stimulation by ionizing radiation or TGFB1. LPAs were reported to induce *COL1A1* mRNA expression in bleomycin- and radiation-induced fibrosis^29^,^30^. We thus hypothesize that the reduction of collagen expression on DGKA inhibition in fibroblasts may at least in part be a result of LPAs as downstream signalling components. We found that DGKA regulates LPA levels and that LPA exposure induced *DGKA* expression in NHDF, again indicating positive feedback loops that could result in fibrosis development in predisposed individuals. The DGKA-mediated change in global levels of signalling lipids after irradiation suggests that epigenetic DGKA regulation should have a profound impact on fibroblast biology and fibrogenesis. We demonstrated these effects by identifying candidate proteins associated with DGKA in fibroblasts. Our partial correlation network analysis as well as earlier reports indicate a DGKA-related, but indirect, interconnection of several PKC isoforms in distinct signalling nodes^46^,^47^,^48^. PKCs are partially regulated by DAGs^5^. Therefore, DAG homeostasis might be co-regulated via both PKC and DGKA in fibroblasts. Conversely, the cellular DAG and PA/LPA equilibrium could be a tool to fine-tune DGKA and PKC downstream signalling. EGR1 was shown to be induced downstream of LPA and PKC^49^, possibly also affecting genomic downstream targets like *DGKA*. The contribution of PRKCA to fibrogenesis has been reported^33^,^50^,^51^. Therefore, we selected PRKCA as a potential co-regulator in DGKA signalling and applied for each kinase a specific inhibitor. We found a synergistic inhibition of cell growth by the two inhibitors in irradiated fibroblasts with low-DGKA methylation, which have been derived from patients developing radiation-induced fibrosis. No synergism was observed in fibroblasts of control patients with high methylation. Additional inhibition experiments showed the cooperation of DGKA and PRKCA in collagen synthesis, another key feature of profibrotic fibroblast activation. We conclude that the effect of DGKA and PRKCA co-inhibition depends on individual traits in patient cells such as epigenetic *DGKA* regulation, pointing out a rationale for individualized therapy in fibrosis.

Promising results have already been obtained using DGKA inhibitors for the treatment of various conditions including cancer^52^ and inflammatory disease^53^. Inhibiting DGKA with small molecules may also prove useful as a preventive antifibrotic therapy. Here we suggest that DGKA inhibition may modulate signalling pathways by altering DAG homeostasis and thus serve as a promising treatment that blocks the profibrotic effects of radiotherapy, especially in patients that show risk-associated *DGKA* methylation.

## Methods

### Study design and patient characteristics

For the present study, 75 breast cancer patients were recruited at the Department of Radiation Oncology, the University Medical Center Mannheim (UMM), Germany. All patients received IORT as a boost to the tumour bed with 20 Gy (INTRABEAM System, 50 kV X-rays; Zeiss Meditec) followed by standard whole breast irradiation (external beam radiotherapy) with doses of 46–50 Gy high-energy X-rays given as 2 Gy per fraction 5 days a week^2^,^54^. Skin biopsies were taken from the inner side of the unirradiated upper arm. The study protocol was approved by the Ethics Committee of the University Hospital of Mannheim. All patients provided informed written consent. Radiation-induced fibrosis of the breast was evaluated by a physician and was defined as grade 2 or higher according to standardized LENT-SOMA^55^ criteria. In more detail, a fibrosis case was defined as a palpable definite increased density and firmness (score 2) or a very marked density or retraction and fixation (score 3) of the irradiated breast 2–5 years after radiotherapy. Compared with score 1 fibrosis with most minor symptoms, these fibrotic breast alterations are of clinical relevance as they are persisting over the observation time and may require clinical intervention. Median follow-up time was 4.9 years (range 2.0–5.5). Treatment-related data were extracted from medical records and epidemiological data were collected from all patients (Supplementary Table 1). None of the characteristics were significantly different between cases and controls.

### Cell culture and reagents

Primary human dermal fibroblast cultures from individual breast cancer patients were established at the UMM as part of the EURATOM/ESTRO GENEPI project^56^ by outgrowth from biopsies, which were taken from the unirradiated skin of the inner upper arm. Medium with 7.5% AmnioMax C-100 supplement (Invitrogen) and 7.5% foetal bovine serum (FBS; Biochrom) was used. The cultures were cryopreserved in the third passage and used for experiments in the fifth to tenth passage. In addition, three NHDF cultures (Promocell) derived from healthy female donors were used. For *in vitro* experiments, fibroblast cultures were grown in fibroblast growth medium 2 (Promocell) supplemented with 50 μg ml^−1^ ascorbic acid and 0.2 mM L-proline. IMR90, BJ and HEK293T cells were cultivated in DMEM/10% FBS. HEK293T cells were obtained from ATCC. IMR90, BJ and HCT116 cells were gifts from the A. Krämer (DKFZ, Heidelberg), J. Hoheisel (DKFZ, Heidelberg) and B. Vogelstein (Ludwig Center, Baltimore) labs, respectively. Cells were irradiated using the ^137^Cs Gammacell 40 Exactor (Best Theratronics) at 1 Gy min^−1^. Cells were routinely tested for absence of mycoplasm contamination using the Venor GeM kit (Minerva Biolabs). HEK293T and HCT116 were verified for cell line identity (Multiplexion GmbH). Treatment with human recombinant TGFB1 (R&D systems) was carried out for the described time points and doses. Bleomycin sulfate, R59949, Ki16425 (Cayman Biochemicals) and further small molecule drugs (Sigma-Aldrich) were dissolved in DMSO.

### Genome-wide DNA methylation analysis

Twenty four patients (12 who developed radiation-induced fibrosis and 12 without fibrosis) were included for genome-wide DNA methylation analysis (Supplementary Table 1) and are a subset of the overall cohort (*n*=75) analysed by MassARRAY. The subset and the overall patient group cohort showed no significant differences (*P* values>0.1, *t*-test for continuous variables, Fisher's exact test for categorical variables). DNA was isolated using the QIAamp DNA mini kit (QIAGEN). Genomic DNA (1.0 μg) was bisulfite treated using the EZ DNA Methylation Gold Kit (Zymo Research) and analysed on the Illumina Infinium HumanMethylation450 Bead Chip Array (Illumina) by the Genomics and Proteomics Core Facility (DKFZ, Heidelberg). The Illumina 450 K array data are available at the European Genome-phenome Archive (EGA accession number EGAS00001001279).

### Quantitative DNA methylation analysis using EpiTYPER

High-resolution DNA methylation analysis was carried out using EpiTYPER MassARRAY technology (Sequenom)^57^. Genomic DNA (1.0 μg) was bisulfite-converted using the EZ DNA methylation kit (Zymo Research). Regions-of-interest were amplified from bisulfite-treated DNA by PCR. Primers (Supplementary Table 8) were designed using EpiDesigner software (Sequenom). Matrix-Assisted Laser Desorption/Ionization-Time of Flight (MALDI-ToF) mass spectrometry was used for quantitative methylation detection and data were analysed with EpiTYPER software 1.2 (Sequenom). Unless stated otherwise, DNA methylation values were calculated as average methylation of all available CpG sites within each PCR product.

### siRNA transfection

siRNA transfection of fibroblasts was carried out using INTERFERin (Polyplus transfection). Cells were transfected using 1.0 μl transfection reagent per 0.02 pmol siRNA and all siRNAs (Dharmacon) were used as a pool of four individual sequences at a combined final concentration of 10 nM.

### mRNA expression analysis using quantitative real-time PCR

Total RNA was isolated using TRIzol (Invitrogen) according to standard protocols. mRNA expression was measured using complementary DNA samples generated from 1.0 μg DNase I-treated RNA with SuperScript III Reverse Transcriptase (Invitrogen) and random hexamers (QIAGEN). Complementary DNA was analysed with a LightCycler 480 real-time PCR system (Roche) and human Universal ProbeLibrary hydrolysis probes (Roche). Relative gene expression was calculated as 2^−ΔΔct^ method with ΔΔct=(ct target−ct housekeeping gene)_treatment_−(ct target−ct housekeeping gene)_control_. Data were normalized to housekeeping gene expression values of beta actin (*ACTB*), glyceraldehyde-3-phosphate dehydrogenase (*GAPDH*) and hypoxanthine phosphoribosyltransferase 1 (*HPRT1*) and the average of the three normalized expression values was taken for individual samples. All primers (Supplementary Table 8) were designed using the Universal ProbeLibrary Assay Design Center application (Roche).

### Cell growth assays

Cell growth was measured using calcein-AM fluorescence assays. Cells were treated with R59949 (5.0 μM), Gö6976 (0.5 μM) or a combination for 48 h and then exposed to ionizing radiation or another co-treatment. Readout was carried out after another 48 h with continued drug treatment by incubating cells seeded in 384-well plates for 60 min with DMEM containing 10% FCS and 0.2 μg ml^−1^ calcein-AM (Sigma-Aldrich). After media removal and washing with PBS cells were lysed in 0.6% Triton X-100/PBS. Viability readout was carried out at 495 nm/520 nm excitation/emission wavelength.

### DNMT activity assay

DNMT activity was determined in nuclear lysates of 1 × 10^6^ patient fibroblasts per sample. Nuclear protein was isolated as described before^58^ and enzymatic activity was measured using the colorimetric DNMT Activity Quantification Kit (Abcam) according to the manufacturer's instruction.

### BrdU incorporation assay

BrdU incorporation assay was carried out using the APC BrdU flow kit (BP Pharmingen) with some modifications. In short, NHDFs were cultivated with R59949 (5.0 μM) and/or Gö6976 (0.5 μM) for 48 h prior to irradiation with 6 Gy. Immediately after that cells were supplemented with BrdU (10 μM) for another 24 h and harvested for permeabilization and staining with APC-coupled anti-BrdU antibody (BD Pharmingen). Staining was carried out at 4 °C overnight and cells were analysed the following day using a FACS Calibur 2 flow cytometer (BD Biosciences).

### Luciferase reporter assays

Genomic regions-of-interest were amplified by PCR from dermal fibroblast DNA and cloned into pGL4.10, pGL4.23 (both Promega) or pCpGfree-promoter-lucia (Invivogen). Reporter constructs were validated by Sanger sequencing (GATC Biotech). Site-directed mutagenesis was carried out according to standard protocols. HEK293T cells were transfected with TransIT-LT1 transfection reagent (Mirus Bio) and readout was carried out 48 h after transfection. Data were normalized to co-transfected luciferase reporter vectors (pRL-TK-renilla luciferase (Promega) for pGL4-based reporters and pGl4-firefly luciferase for pCpGfree-lucia reporters). *In vitro* methylation of reporters was carried out using M.SssI CpG methyltransferase (Thermo Scientific).

### Quantification of total hydroxyproline

Hydroxyproline in cell culture media was measured based on a modified protocol for high-performance liquid chromatography (HPLC) with pre-column derivatization^59^. Total protein from cell culture media (3.0 ml) was hydrolysed overnight in 6 M hydrochloric acid, neutralized, buffered with sodium borate (pH 9.6) and derivatized using *o*-phthaldialdehyde (Sigma-Aldrich) and subsequently 9-fluorenylmethoxycarbonyl chloride (FMOC-Cl, Sigma-Aldrich). Samples were extracted twice with diethyl ether and analysed on a 1100 series HPLC system (Agilent) with a LiChrospher 100 RP18 column (Agilent) and a 1046A fluorescence detector (Hewlett Packard). Hydroxyproline signals were normalized to sarcosine (8.5 μM) added as an internal standard before acid hydrolysis and signals were corrected for cell number determined with a CASY cell counter (Roche).

### DAG kinase assay

DAG kinase assay was carried out using the ADP-Glo kinase assay (Promega) as previously described^25^ in 384-well plates. In short, samples were lysed in 2 μl lysis buffer (50 mM HEPES, pH 7.2, 150 mM NaCl, 5 mM MgCl_2_, 1 mM dithiothreitol, 1 mM PMSF and protease inhibitor), followed by addition of 8 μl reaction buffer (to a total concentration of 50 mM MOPS, pH 7.4, 50 mM *n*-octyl β-D-glucopyranodie (Sigma-Aldrich), 1 mM dithiothreitol, 100 mM NaCl, 20 mM NaF, 10 mM MgCl_2_, 1 μM CaCl_2_, 10 mM phosphatidylserine (Sigma-Aldrich), 2 mM 1,2-dioleoyl-sn-glycerol (Sigma-Aldrich), 0.2 mM ATP). After a 30-min reaction at 30 °C, 10 μl ADP-Glo reagent (Promega) were added and after 40 min at room temperature 20 μl of Kinase Detection Reagent (Promega) was added. Luminescence readout was carried out after an additional 40 min at room temperature. Luminescence values were normalized to cell viability determined in matched experiments using a calcein-AM fluorescence assay.

### Lipid extraction and UPLC-ESI-MS/MS analysis

DAGs were extracted from 3 × 10^4^ NHDF per sample using previously described protocols^26^. Briefly, cells were harvested in cold methanol/chloroform/12 M HCl (400:400:3). About 50 ng of 1,3-diheptadecanoyl glycerol-*d5* (Avanti Polar Lipids) were added as an internal standard for DAGs prior to extraction. Lipid extracts were reconstituted in MeOH/H_2_O (95:5 v/v)+1 mM ammonium formiate/0.05% formic acid and subjected to UPLC-ESI-MS/MS analysis^60^.

For the quantification of (L)PAs, extracts were prepared as described for DAGs, dissolved in chloroform/methanol/water (30:60:8) and were purified through an anion-exchange column using a diethyl-aminoethyl resin (DEAE-Sephadex). The acidic fraction was then subjected to a reverse phase chromatography with C18 columns to remove all salts from the final extracts. Those were re-dissolved in 80% methanol containing 25 ng of 1-hexadecanoyl(d31)-2-(9Z-octadecenoyl)-sn-glycero-3-phosphate (Avanti Polar Lipids) used as an internal standard prior to UPLC-ESI-MS/MS analysis. Both DAG and (L)PA signals were corrected for cell number determined with a CASY cell counter.

Reversed-phase chromatography was carried out on a Waters ACQUITY I class UPLC system with either a Acquity UPLC BEH or a CSH column, for DAG or (L)PA analyses, respectively. Both 1.7 μm C18 columns were of 50 × 2.1 mm and 130 Å in size, and were coupled to a Waters Xevo TQ-S tandem mass spectrometer. The gradient used to separate DAGs has been published before^60^, whereas (L)PAs were separated using the gradient described in Supplementary Table 4. Specifc lipid species were detected and quantified using multiple reaction monitoring with a cone voltage set to 50 V, whereas the collision energy varied from 14 eV for the detection of DAGs to 20 and 35 eV for LPA and PA detection, respectively. Annotations of different DAGs were based on *m/z* transitions of the ammonium adduct of the parent mass ([M+NH_4_]^+^) to the respective monoacylglycerol (MAG)-daughter ions missing one fatty acid and ammonia ([MAG+H]^+^). For DAGs containing two different fatty acids, both transitions (loss of FA1 and NH_3_→[MAG1+H]^+^ and loss of FA2 and NH_3_→[MAG2+H]^+^) were monitored and peak areas of both transitions were summed (Supplementary Table 5). (L)PAs were quantified using the fragment corresponding to the loss of the glycerol backbone and the phosphate (*m/z* 152.7, Supplementary Table 6). DAG amounts were expressed as relative DAG amount per lipid extract and were obtained from the ratio of the peak areas of the respective DAGs to the peak area of the internal standard. Similarly, respective (L)PAs were normalized to the internal standard and were expressed as relative to the total amount of (L)PA.

### Chromatin immunoprecipitation

Cells were crosslinked with 1.0% formaldehyde, harvested and resuspendend in swelling buffer (25 mM HEPES-KOH pH 7.8, 2 mM MgCl_2_, 10 mM KCl, 0.1% v/v nonidet-P40, 0.5 mM PMSF and protease inhibitor). Nuclei were collected by centrifugation and lysed in sonication buffer (10 mM Tris-HCl, pH 8.0, 200 mM NaCl, 1 mM EDTA, 0.5% m/v *N*-lauroylsarcosine, 0.1% sodium deoxycholate and protease inhibitor) before sonication with a Covaris S2 Sonicator (Covaris). For ChIP of histone modifications, 2 × 10^5^ cells and for EGR1 and FOS 3 × 10^6^ cells were used. ChIP was conducted using the SX-8G IP-Star Automated System (Diagenode). Antibodies against H3K4me3 (pAb-003-050, Diagenode, 1:100 dil.), H3K27me3 (pAb-195-050, Diagenode, 1:100 dil.), H3K27ac (39133, Active Motif, 1:100 dil.), H3K4me1 (ab8895, Abcam, 1:100 dil.), FOS (9F6, Cell Signaling, 1:40 dil.) and EGR1 (15F7, Cell Signaling, 1:40 dil.) were used. Subsequent quantification was run on a LightCycler 480 with PCR primers for Universal ProbeLibrary (Supplementary Table 8). Signals were normalized to non-immunoprecipitated chromatin controls (input).

### Chromatin conformation capture

3C sample preparation and data analysis were carried out as described previously^61^. About 1 × 10^6^ cells were crosslinked in 2.0% formaldehyde, harvested and DNA was digested using CviQI (New England Biolabs) overnight. Relative crosslinking frequency was analysed using quantitative PCR with primers for Universal ProbeLibrary hydrolysis probes (Supplementary Table 8). Normalization was carried out using primers for quantification of genome equivalents and crosslinking frequency at a region showing uniform crosslinking frequency in different samples.

### Western blot analysis

Total protein was isolated by lysing cells in RIPA lysis buffer (50 mM Tris-HCl pH 8.0, 150 mM NaCl 1.0% (v/v) nonidet-P40, 0.5% m/v sodium deoxycholate, 0.1% m/v sodium dodecyl sulfate) supplemented with protease inhibitor (Roche). Western blot analysis was run with NuPAGE SDS-PAGE gel system (Life Technologies) and primary antibodies against DGKA (11547-1-AP, Proteintech), beta actin (ACTB; sc-47778 HRP, Santa Cruz Biotechnology), PRKCA (2056P, Cell Signaling Technology), collagen 1 (EPR7785, Abcam) and appropriate secondary antibodies (Santa Cruz Biotechnology). Detection was carried out using ECL reagent (Perkin-Elmer) and uncropped western Blot images are provided in Supplementary Figs 4,16 and 21. Band intensities were quantitated with ImageJ software using beta actin band intensities for normalization.

### LC-MS-based protein quantification

Fibroblasts were lysed using RIPA buffer before subjecting to sonication (10% amplitude) and one freeze–thaw cycle. About 100 μg protein (quantified by Pierce bicinchoninic acid Protein Assay Kit, Thermo Scientific) were precipitated with chloroform/methanol^62^. Samples were resolubilized in tryptic digestion buffer (50 mM Tris-HCl, pH 8.0, 1 mM CaCl_2_) and digested with Trypsin (1:30, w/w) for 15 h at 37 °C. Following acidification, digests were desalted using a Peptide Desalting Lab-in-a-Plate Flow-Thru–plate (C18, Glygen), dried and resuspended in 3% acetonitrile, 0.1% formic acid, 0.01% TFA in water containing the heavy peptide pool (20 fmol μl^−1^) synthesized with heavy isotopic lysine or arginine at the C terminus (JPT).

SRM analysis was performed on a QTRAP 6500 mass spectrometer (AB SCIEX) operated with Analyst software (v1.6.2) and coupled to a nanoAcquity UPLC (Waters). Reversed-phase chromatography was performed on a Acquity UPLC M-Class CSH C18 column (300 μm × 15 cm, 130 Å) (Waters). Samples were separated over 120 min at a flow rate of 6 μl min^−1^ using 4–30% (1–110 min), 30–85% (110–120 min) acetonitrile gradient in 0.1% formic acid, 0.01% TFA.

For SRM method optimization and validation, MS/MS spectra were acquired in the trap mode (enhanced product ion) with dynamic fill time, Q1 resolution low, scan speed of 10,000 Da s^−1^, *m/z* range of 100–2,000. The best two transitions for each peptide were selected based on maximum signal intensities. For the final SRM quantification experiment, two reproducibly detectable peptides per protein with at least two charges were targeted with two transition signals per heavy or light peptide (Supplementary Data 1). This resulted in a total of 192 transitions for the 48 peptides deriving from 24 proteins. Scheduled SRM was performed with Q1 operated in unit resolution, Q3 in low resolution, a target scan time of 1.5 s, a minimal dwell time of 58 ms and retention time windows of ±1.5 min around the specific elution time.

### mRNA sequencing

mRNA next generation sequencing (RNA-seq) was carried out using 5 μg of total genomic RNA from NHDF derived from a healthy female donor NHDF (Promocell). The sequencing library was prepared using NEBNext Ultra Directional library preparation reagents (New England Biolabs). Sequencing and sequence alignment were carried out at the Genomics and Proteomics Core Facility (German Cancer Research Center, Heidelberg, Germany). Data were mapped to human genome (GRCHh37/hg19).

### ChIP sequencing

DNA libraries were prepared from 2 to 5 ng of immunoprecipitated DNA using NEBNext Ultra DNA library Prep Kit (New England Biolabs). Sequencing was carried out at the Genomics and Proteomics Core Facility (German Cancer Research Center, Heidelberg, Germany). Data were mapped to human genome (GRCHh37/hg19) using Bowtie^63^ and peaks were called using MACS^64^.

### Statistics

Results show mean and s.e.m. unless indicated otherwise. Two-tailed Student's *t*-test was used and results with *P* value <0.05 were considered as statistically significant. The Holm–Sidak method was used to correct for multiple testing in settings of multiple *t*-tests. Methylation levels were compared by the Wilcoxon rank-sum test assuming a non-parametric distribution. Linear correlation was assessed using the Pearson correlation coefficient (*r*). *P* values for linear regression refer to significance of slope deviation from zero determined by F test. Data were visualized with GraphPad Prism version 5 (GraphPad Software). Clustering and heatmap graphs were generated using MultiExperiment Viewer (TM4) with Pearson's correlation and average linkage settings. Drug treatment synergy was determined using the original Bliss independence criterion as described before^35^. Transcription factor-binding sites were predicted using TRANSFAC, JASPAR, PROMO and ConSite (Supplementary Table 3).

For identification of significant DNA methylation differences, filtering of raw data from the array evaluation consisted of: (i) filtering all beta values with the number of beads less than 3 and setting the corresponding detection *P* values equal to 1; (ii) setting all beta values with a detection *P* value >0.01 to missing; (iii) excluding all probes with a proportion of missings >0.3; (iv) excluding SNP-associated probes. Preprocessing included: (i) missing values imputation performed by applying the *k*-nearest neighbour method^65^ with *k*=10; (ii) colour bias normalization via smooth quantile normalization, applied for adjustment of intensities measured in two colour channels of the Illumina chip; (iii) beta-mixture quantile normalization^66^. All probes with a s.d. of mean methylation levels <5% were excluded from the further analysis. Statistical analysis was performed to detect differentially methylated CpG sites by use of multivariable beta regression with explanatory variables fibrosis status (yes/no), patient's age at the date of sample acquisition, smoking status (ever smoker: yes/no) and exposure to chemotherapy at the date of sample acquisition (yes/no), resulting in a list of *P* values of the corresponding two-sided Wald tests for testing the effect of fibrosis on beta values. The Benjamini–Hochberg method was then used to adjust for multiple testing to control the false discovery rate (FDR) at 5% level.

SRM data were processed using the Skyline software (v2.6.0). The default peak boundary assignment based on Savitzky–Golay smoothing was manually reassigned if required. For each peptide, peak areas of corresponding transitions were summed for analysis. The ratio between the background-reduced peak area of the light transition and the background-reduced peak area of the heavy transition was calculated to correct for ionization or spray differences between runs.

Partial correlation analysis of DGKA with associated proteins was carried out applying the Graphical Gaussian Model approach as proposed previously^67^. Two-sided *P* values for estimated partial correlations, as well as posterior probabilities for each edge, that is, the empirical posterior probabilities for an edge to be present, were computed. Defining the local FDR^32^ for each edge as 1—posterior probability and setting an upper bound for the posterior probability equal to 0.6, we selected the edges to be included into the final network. All statistical analyses were performed by using the statistical software environment R, Version 3.0.2 with R-packages impute, betareg and GenNet and Bioconductor packages lumi and methylumi.

## Additional information

**Accession codes:** The Illumina 450 K array data have been deposited in the the European Genome-phenome Archive (EGA) under the accession number EGAS00001001279.

**How to cite this article:** Weigel, C. *et al*. Epigenetic regulation of diacylglycerol kinase alpha promotes radiation-induced fibrosis. *Nat. Commun.* 7:10893 doi: 10.1038/ncomms10893 (2016).

## Supplementary Material

Supplementary Information Supplementary Figures 1-22, Supplementary Tables 1-8 and Supplementary References

Supplementary Data 1 Peptide transition signals used for quantitative protein mass spectrometry
