# Supplementary material for: Epigenetic regulation of diacylglycerol kinase alpha promotes radiation-induced fibrosis
Source: Nat Commun. 2016 Mar 11;7:10893. doi: 10.1038/ncomms10893 (PMC4792958; doi:10.1038/ncomms10893)
Supplement: Supplementary Information — Supplementary Figures 1-22, Supplementary Tables 1-8 and Supplementary References [file ncomms10893-s1.pdf]

Supplementary information

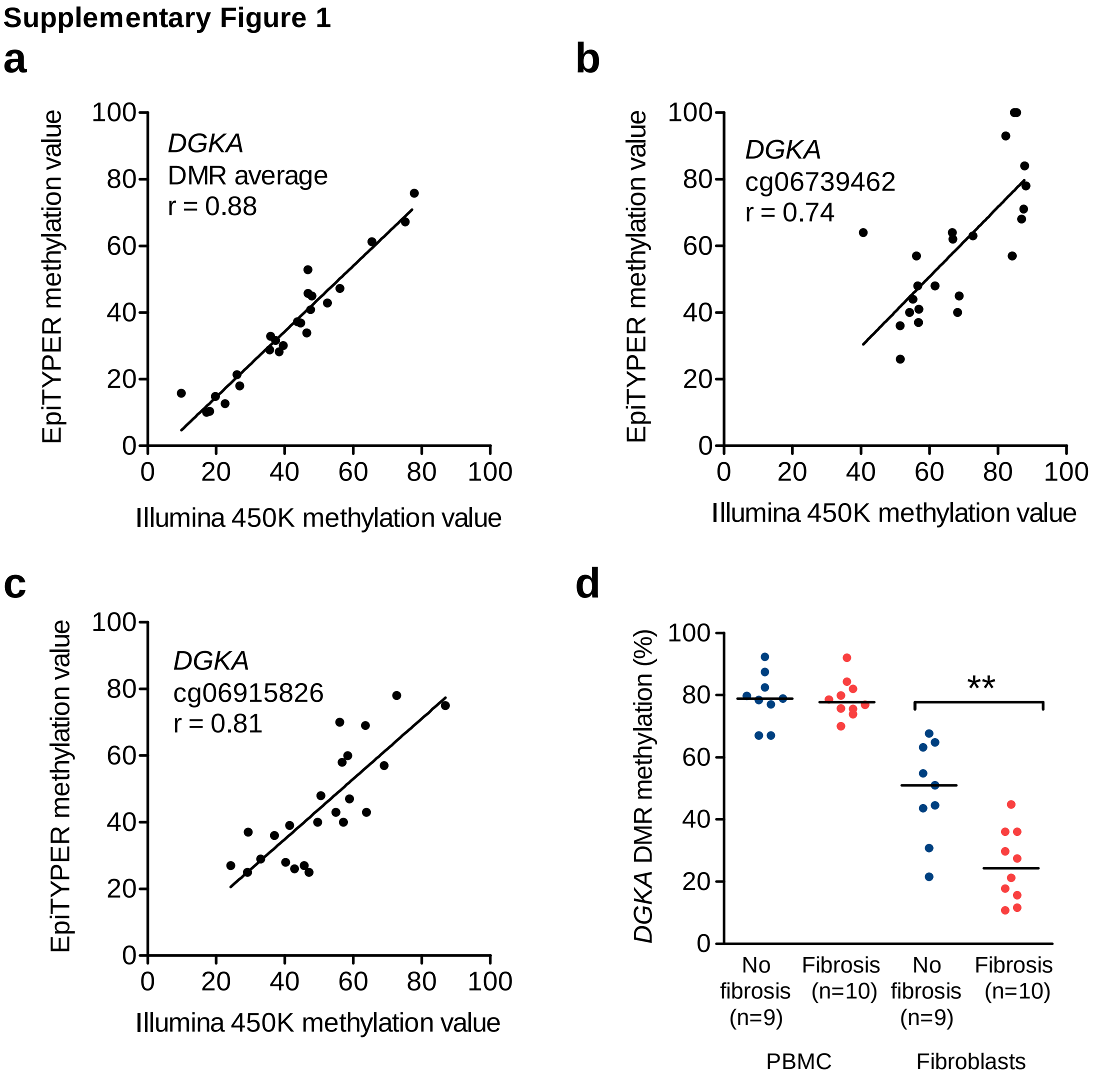

Supplementary Figure 1. Validation of DNA methylation at the *DGKA* differentially methylated region (DMR) using EpiTYPER technology. Validation of DNA methylation across the *DGKA* DMR was carried out comparing mean methylation of all detectable CpG units at the DMR to all overlapping Illumina Infinium probes (**a**) or single CpG site comparison of the two differentially methylated CpG probes cg06739462 (**b**) and cg06915826 (**c**). Data show linear correlation and Pearson correlation coefficients (r) using 48 individual primary fibroblast samples. (**d**) Investigation of *DGKA* DMR methylation in a subset of 19 fibroblasts and matched PBMC either belonging to a cohort that developed fibrosis (Fibrosis) or a fibrosis-free control group (No fibrosis). Data show individual values from fibroblast and groupmedian. ** $p<0.01$, Wilcoxon rank-sum test.

Supplementary Figure 2

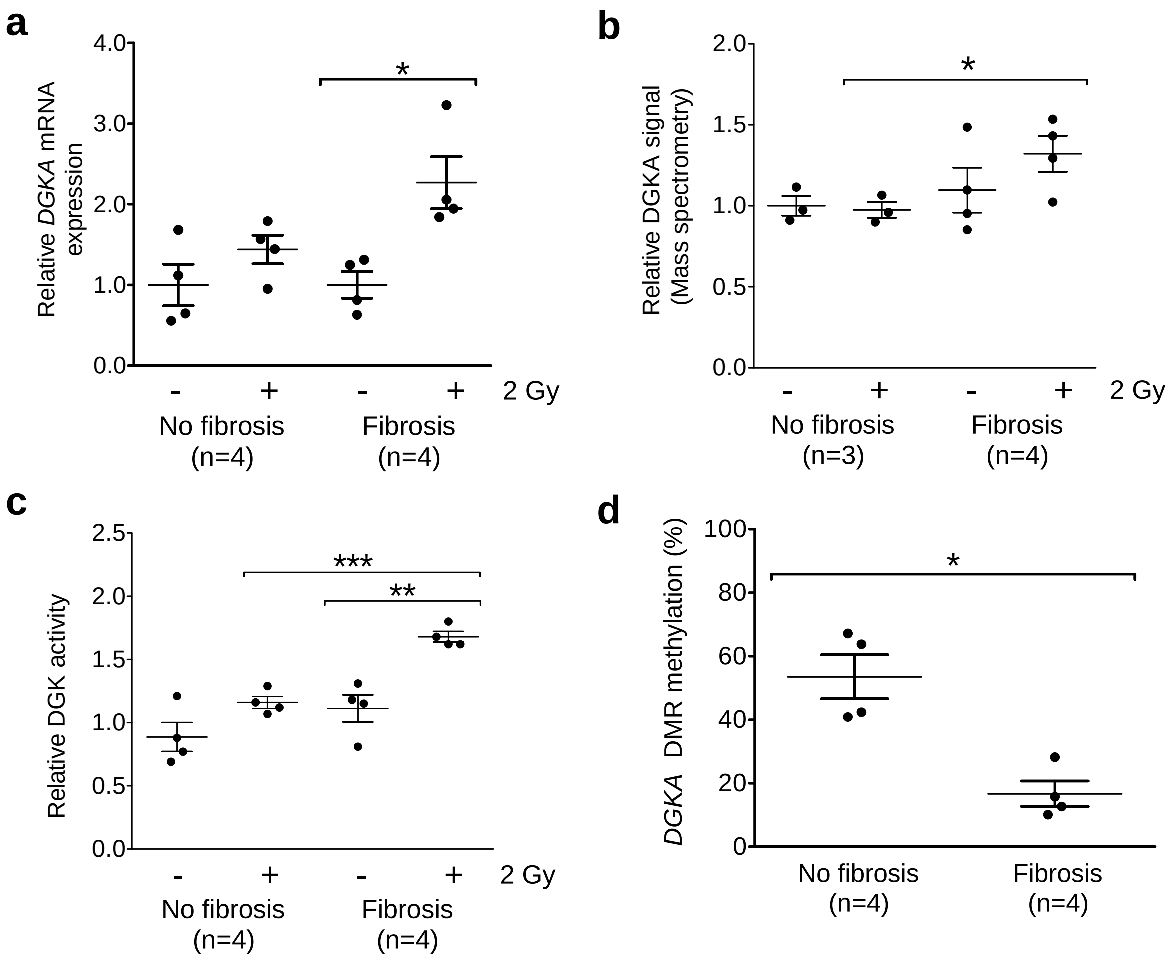

Supplementary Figure 2. Comparison of *DGKA* induction and DMR methylation in patient-derived fibroblasts shows high DGKA induction in patients with increased fibrosis risk. *DGKA* induction in a subgroup of fibroblasts from patients who developed radiation fibrosis (Fibrosis, n=4) was compared to a control group derived from patients that showed no fibrosis (No fibrosis, n=4; n=3 for mass spectrometry). Data show *DGKA* mRNA expression (**a**), protein expression determined by mass spectrometry (**b**), differences in diacylglycerol kinase (DGK) activity (**c**) and DNA methylation differences at the *DGKA* DMR (**d**). Fibroblasts were studied untreated or after 2 Gy radiation. Dots show mean values from duplicate experiments for each fibroblast (triplicates for (C)), bars depict mean ± SEM in each group. * $p<0.05$, ** $p<0.01$, *** $p<0.001$, using t test (A-C) and Mann-Whitney test (D).

Supplementary Figure 3

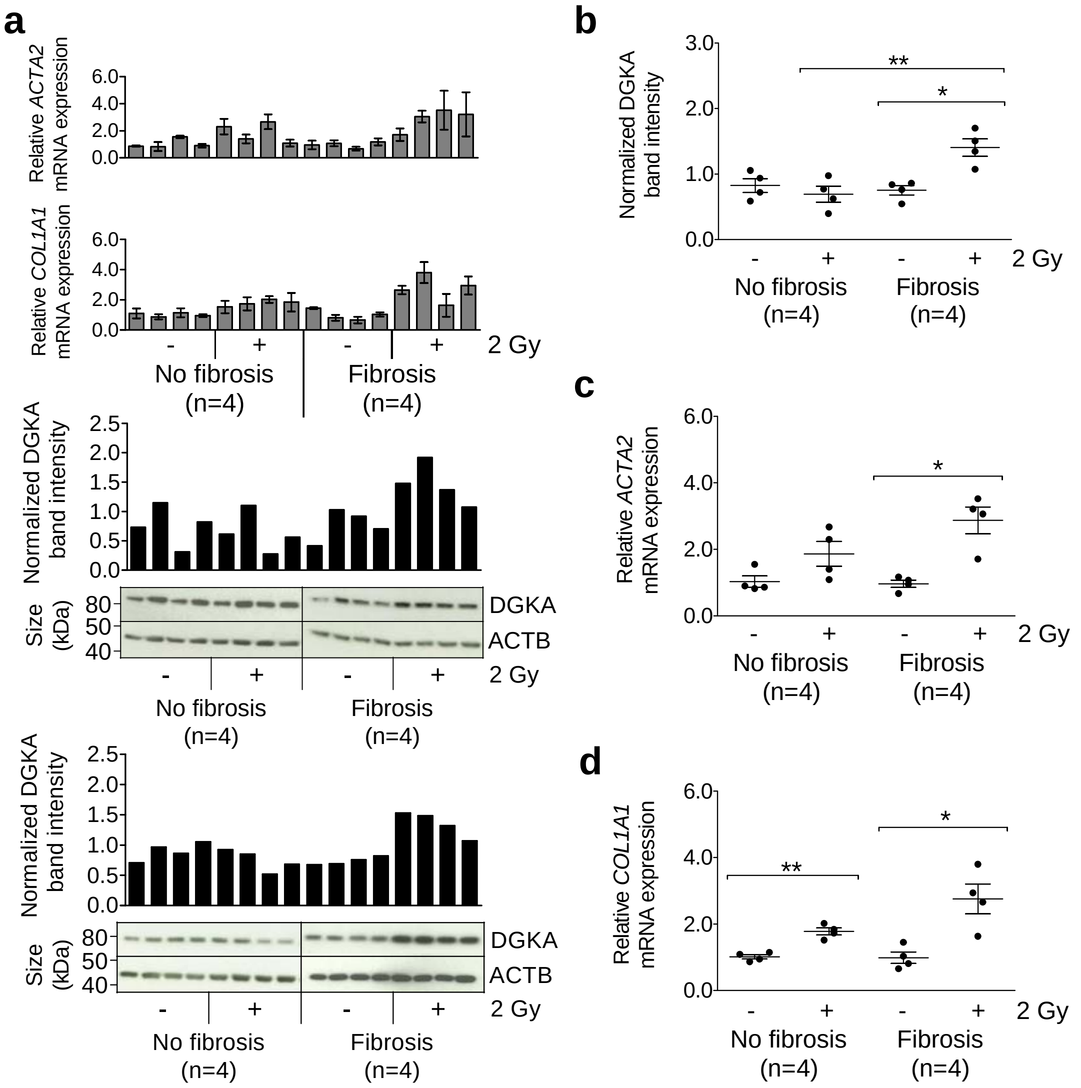

Supplementary Figure 3. Inducibility of DGKA in fibroblasts from patients developing radiation-induced fibrosis leads to increased DGKA protein levels. (**a**) mRNA expression and Western blot analysis carried out in patient fibroblasts (n=8) 48h after ionizing radiation (2 Gy) or mock treatment. Data show bands detected for beta actin (ACTB) and DGKA with normalized band intensities in two independent replicate experiments. Western Blot images have been cropped to highlight specific bands. Matched mRNA expression values of *COL1A1* and *ACTA2* have been added. Bars show mean±SEM from triplicate experiments (**b**) Analysis of DGKA by Western blot in patient fibroblasts (n=8). (**c,d**) Group-wise comparison of *ACTA2* (c) and *COL1A1* (d) mRNA expression in patient fibroblasts. Data show mean±SEM for each group. *p<0.05, **p<0.01, Student's t test.

Supplementary Figure 4

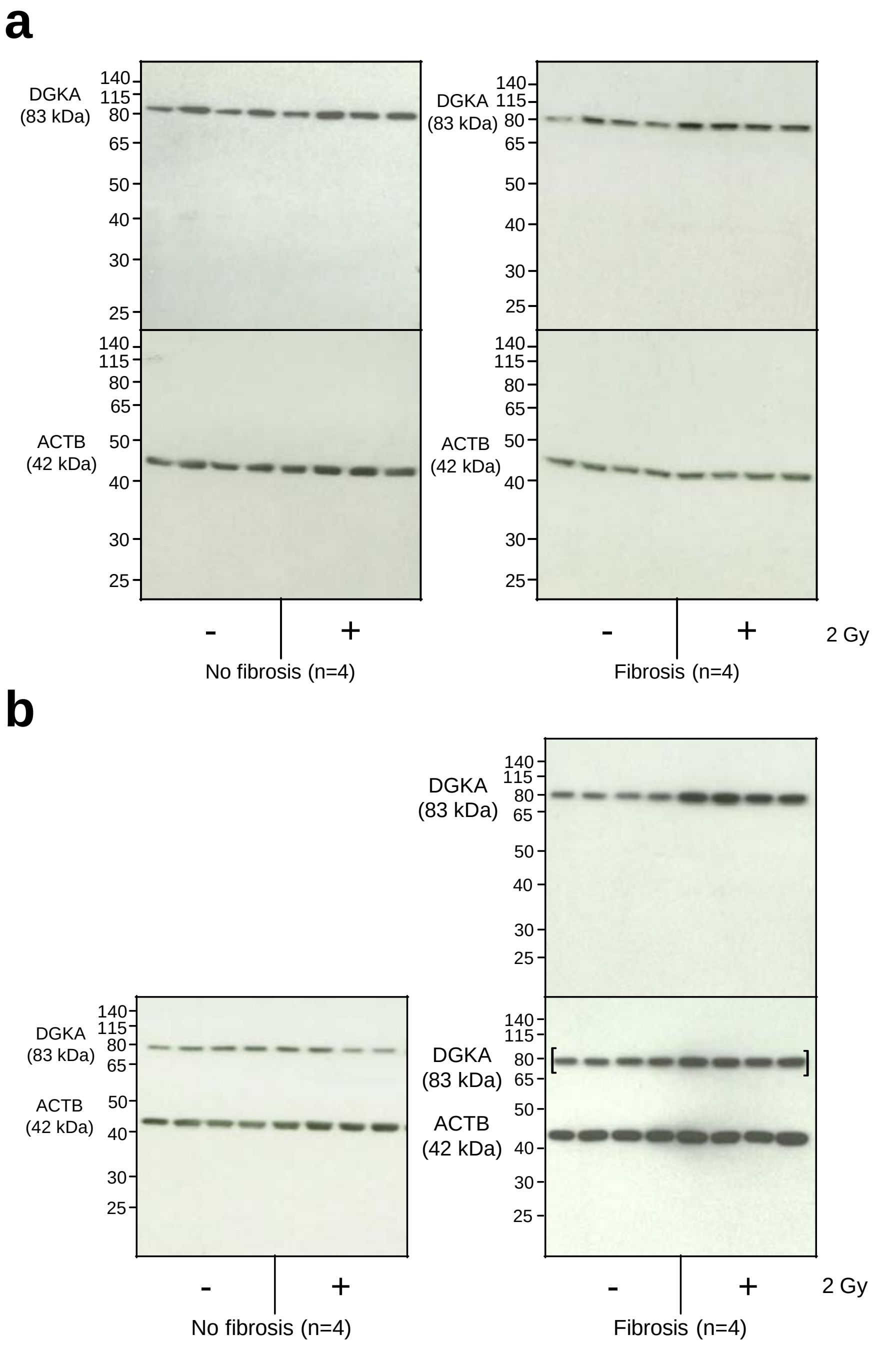

Supplementary Figure 4. Uncropped Western Blot images for DGKA detection in patient fibroblasts (n=8). Graph shows uncropped images of DGKA and ACTB-stained Western Blot images from Supplementary Figure 3a. Duplicate experiments (**a,b**) are shown. Proteins and corresponding sizes are indicated. Size ladders (left) show marker size in kDa. Co-stained protein bands (in brackets) were not used for quantitative analysis in Supplementary Figure 3b.

Supplementary Figure 5

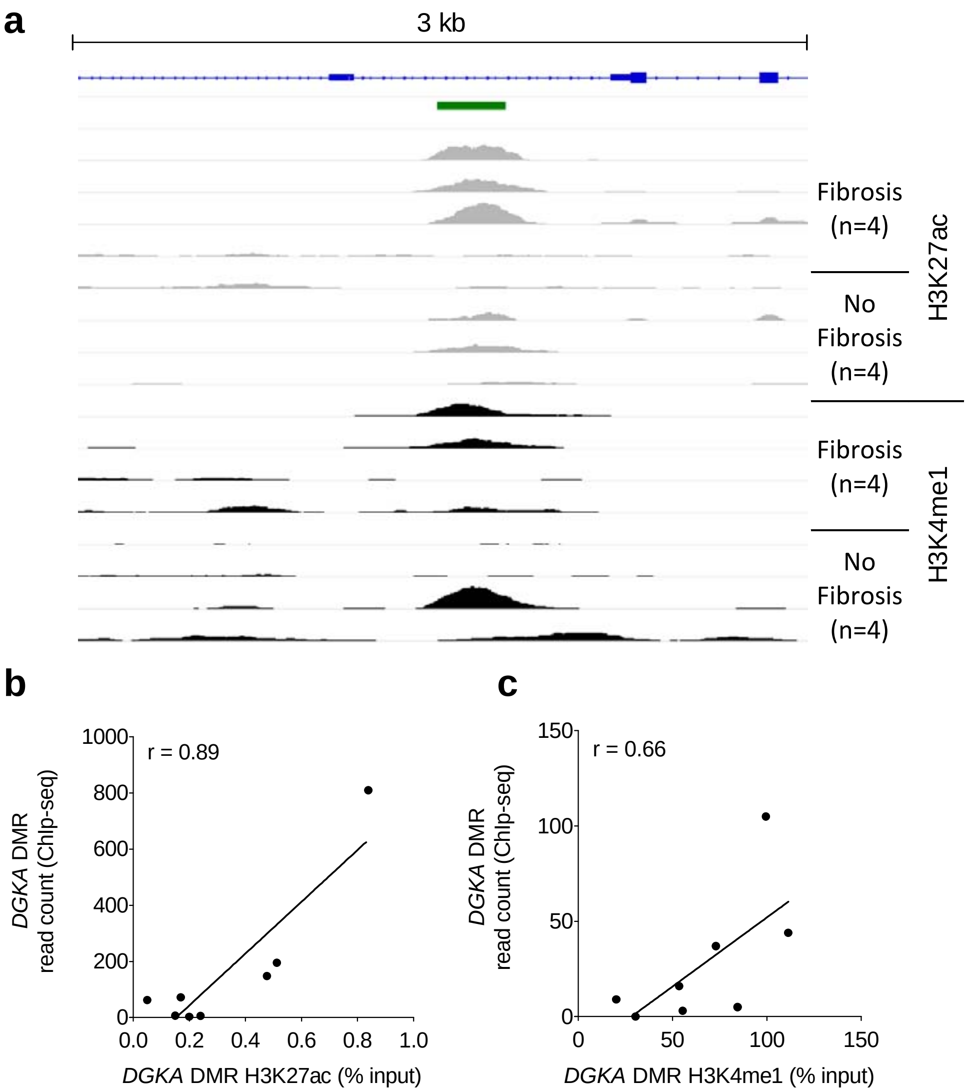

Supplementary Figure 5. Validation of *DGKA* DMR histone marks with ChIP-seq signals generated from patient fibroblasts. (**a**) ChIP-seq signal tracks of 8 patient fibroblast samples for H3K27ac (grey) and H3K4me1 (black). DMR CpG island (green) and *DGKA* gene transcript (blue) are shown. (**b,c**) Linear correlation of read counts obtained from ChIp-seq and relative signal intensities obtained from qPCR for H3K27ac (**b**) and H3K4me1 (**c**) at the *DGKA* DMR. Pearson correlation coefficients (r) are indicated.

Supplementary Figure 6

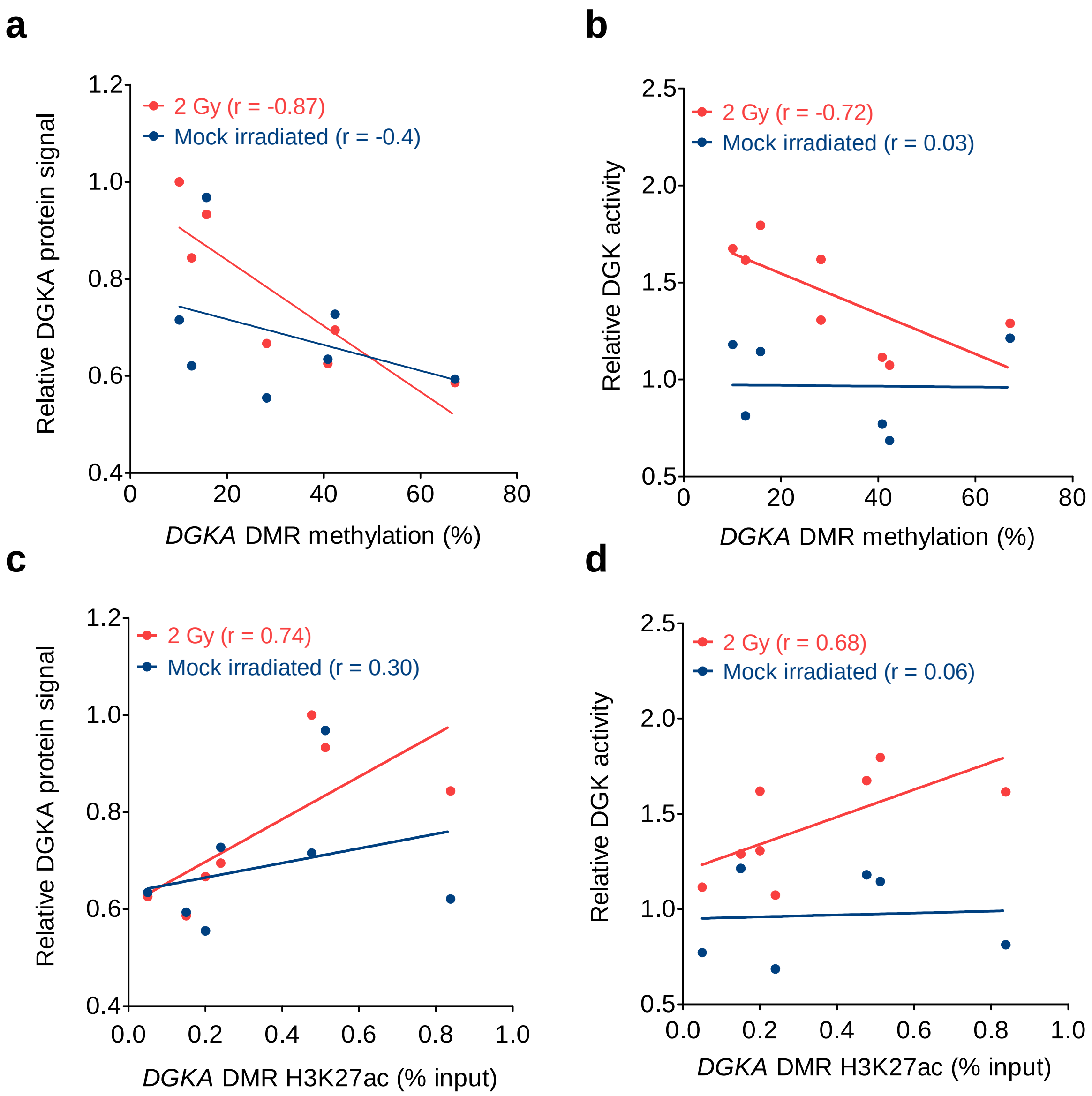

Supplementary Figure 6. DNA methylation and histone H3K27ac at the *DGKA* DMR correlate with inducible DGKA protein and DGK activity in patient-derived fibroblasts. Average DNA methylation across the *DGKA* DMR obtained from the EpiTYPER assay (**a,b**) and H3K27ac signal obtained via chromatin immunoprecipitation (**c,d**) were correlated with patient fibroblast (n=7) DGKA amount measured by quantitative mass spectrometry or determination of global DGK activity. Data are shown for fibroblasts after ionizing radiation (2 Gy) (red dots) or controls (blue dots), and corresponding Pearson correlation coefficients (r) are indicated.

Supplementary Figure 7

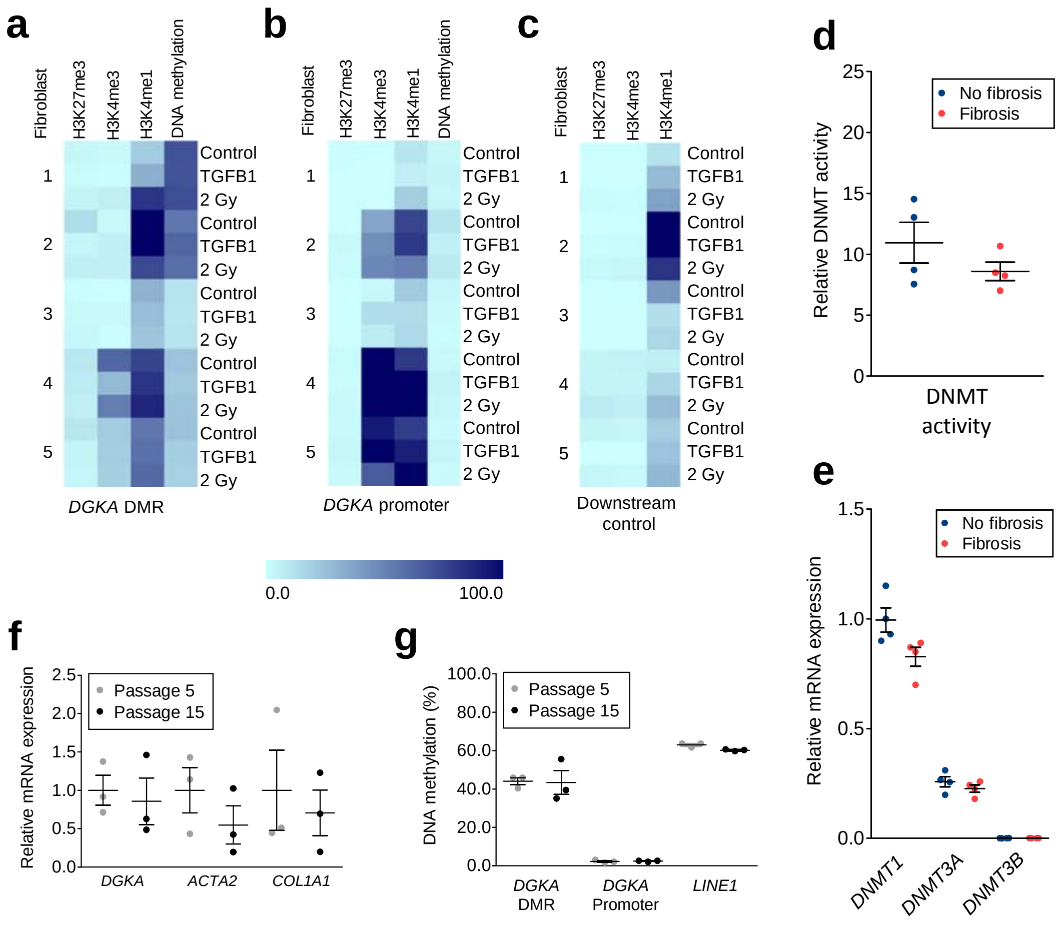

Supplementary Figure 7. Heterogeneity and robustness of epigenetic regulation at the *DGKA* *locus* in fibroblasts. Heatmaps showing relative histone modification abundance (% input DNA) from chromatin immunoprecipitation experiments and DNA methylation from EpiTYPER assay. Data were derived from 5 individual patient-derived fibroblasts treated for 120h with TGF-beta 1 (4 ng ml^{-1}) or 72 h after single dose gamma radiation (2 Gy) exposure. The DGKA DMR (**a**), promoter (**b**) and a 3'intragenic control region (**c**) were investigated. Scale bars indicate % input DNA for ChIP data and % DNA methylation for EpiTYPER derived from duplicate experiments. (**d,e**) DNA methyltransferase (DNMT) activity (**d**) and mRNA expression of *DNMT1*, *DNMT3A* and *DNMT3B* (**e**) in patient fibroblasts from either the fibrosis or non-fibrosis cohort (n=4 each). Data show mean±SEM of triplicate experiments per fibroblast strain. (**f,g**) Monitoring of mRNA expression (**f**) and DNA methylation (**g**) in early (Passage 5) and late (Passage 15) passage fibroblasts. *DGKA* promoter site refers to the *DGKA* promoter CpG island (DGKA_Promoter_2 in Supplementary Table 8). Data depict mean±SEM from duplicate experiments in NHDF (n=3).

Supplementary Figure 8

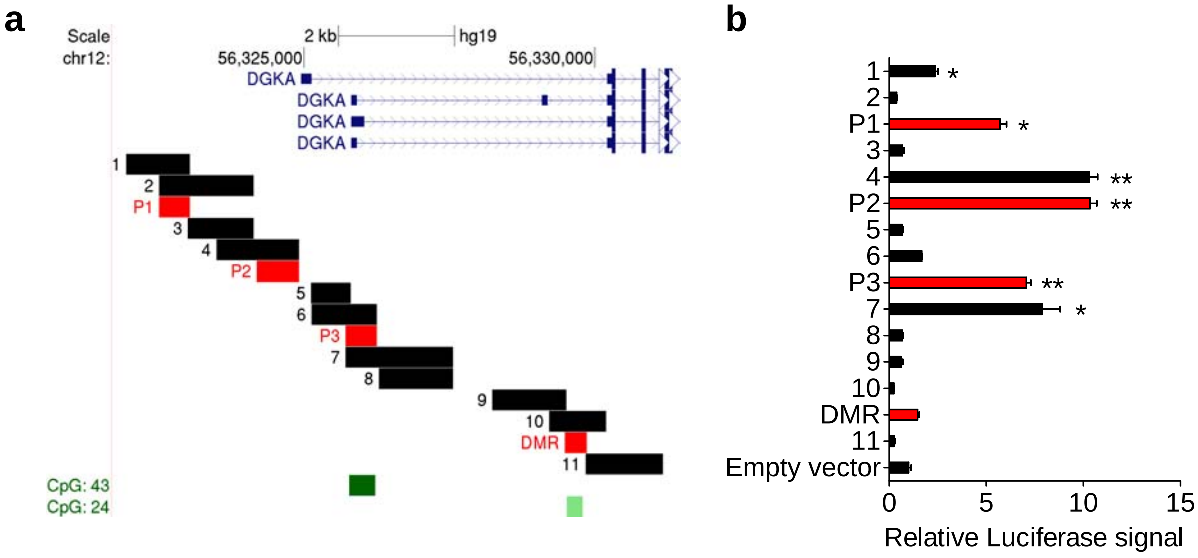

Supplementary Figure 8. Profiling of the *DGKA* 5‘untranslated region (UTR) in a dual luciferase reporter assay reveals three sites of promoter activity. (**a**) Map of the *DGKA* 5‘UTR with DGKA transcripts (blue bars), CpG islands (green bars) and luciferase reporter constructs using pGl4.10 reporter vectors in HEK293T cells (red bars represent constructs presented in Fig. 2 D and E). (**b**) Corresponding relative luciferase signals normalized to internal renilla luciferase control and empty vector background signal. Data depict mean ± SEM from at least 4 individual replicates. * $p<0.05$, ** $p<0.01$ compared to empty vector, Student’s t test.

Supplementary Figure 9

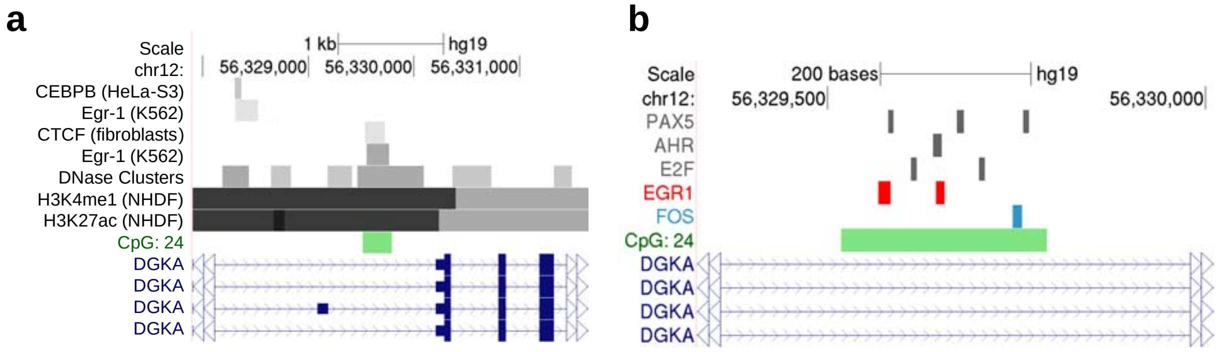

Supplementary Figure 9. Transcription factor binding sites at the *DGKA* DMR. (**a**) ChIP-seq data from the ENCODE project[1] at the DGKA DMR derived from adult NHDF, fibroblasts, HeLa-S3 or K562 cells. Grey bars indicate ChIP-seq and DNAse-seq peaks, with darker colors showing higher peak intensity. (**b**) Location of the transcription factor binding motives predicted by at least two out of four algorithms (see Supplementary table 3) at the *DGKA* DMR CpG island are shown as red, blue and grey bars. CpG islands (green) and *DGKA* transcript (blue) have been added.

Supplementary Figure 10

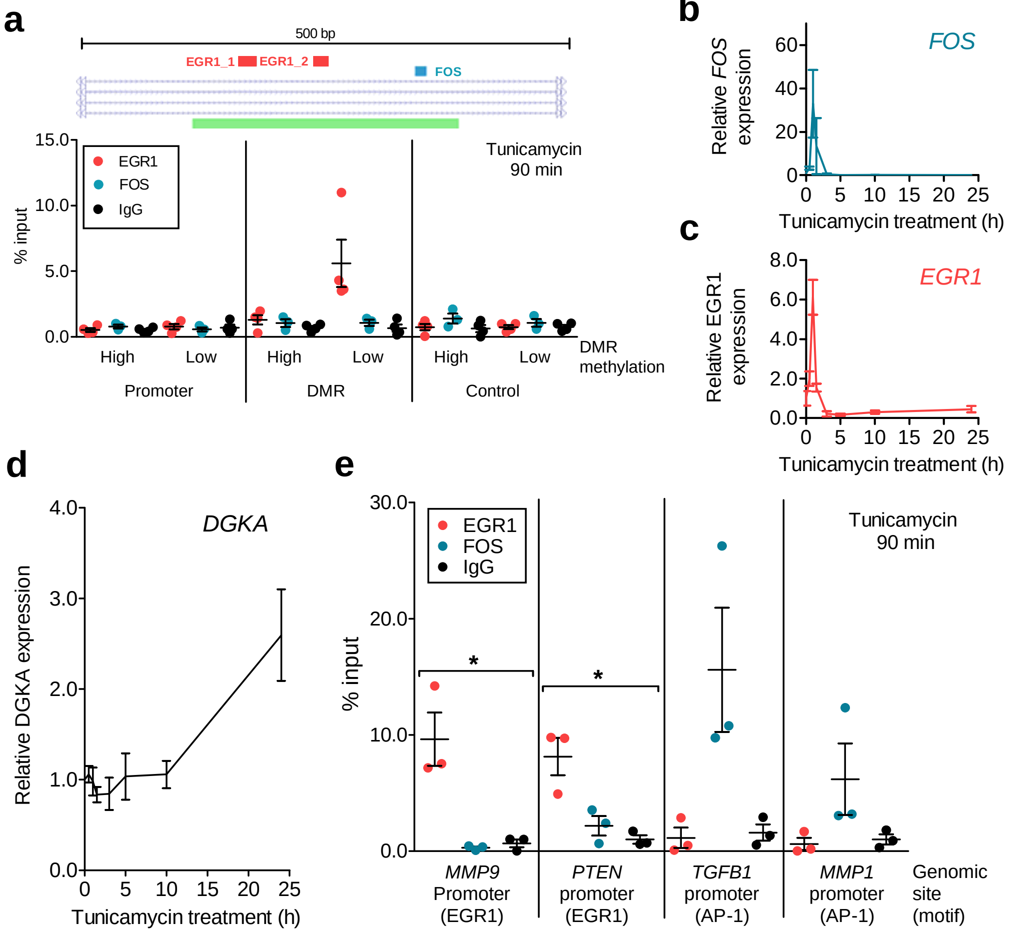

Supplementary Figure10. Investigation of transcription factor binding at the *DGKA* locus under stress factor stimulation. (**a**) Upper panel: Map of the DGKA DMR with CpG islands (green), predicted FOS (blue), EGR1 (black) binding sites and PCR amplicon for ChIP-qPCR (red). Lower panel: Primary human dermal fibroblasts with differential DNA methylation at the *DGKA* DMR (high methylation, n=4; low methylation, n=4) were exposed to the profibrotic and stress mimetic compound tunicamycin[2], an inductor of FOS expression, (0.75 µM for 90 min). Chromatin immunoprecipitation for FOS, EGR1 or rabbit IgG (control) at the *DGKA* DMR, DGKA promoter or an intragenic control region are shown. Dots show mean values from 4 independent immunoprecipitatins per fibroblast sample, bars depict mean ± SEM in each group. (**b,c,d**) mRNA induction of *FOS* (**b**), *EGR1* (**c**) and *DGKA* (**d**) after tunicamycin exposure for up to 24 h. (**e**) Verification of EGR1 (*MMP9*[3] and *PTEN*[4] promoter) and FOS/AP-1 (*TGFB1*[5] and *MMP1*[6] promoter) binding at previously reported transcription factor binding sites. Data (b-e) depict mean ± SEM from duplicate experiments in NHDF (n=3).

Supplementary Figure 11

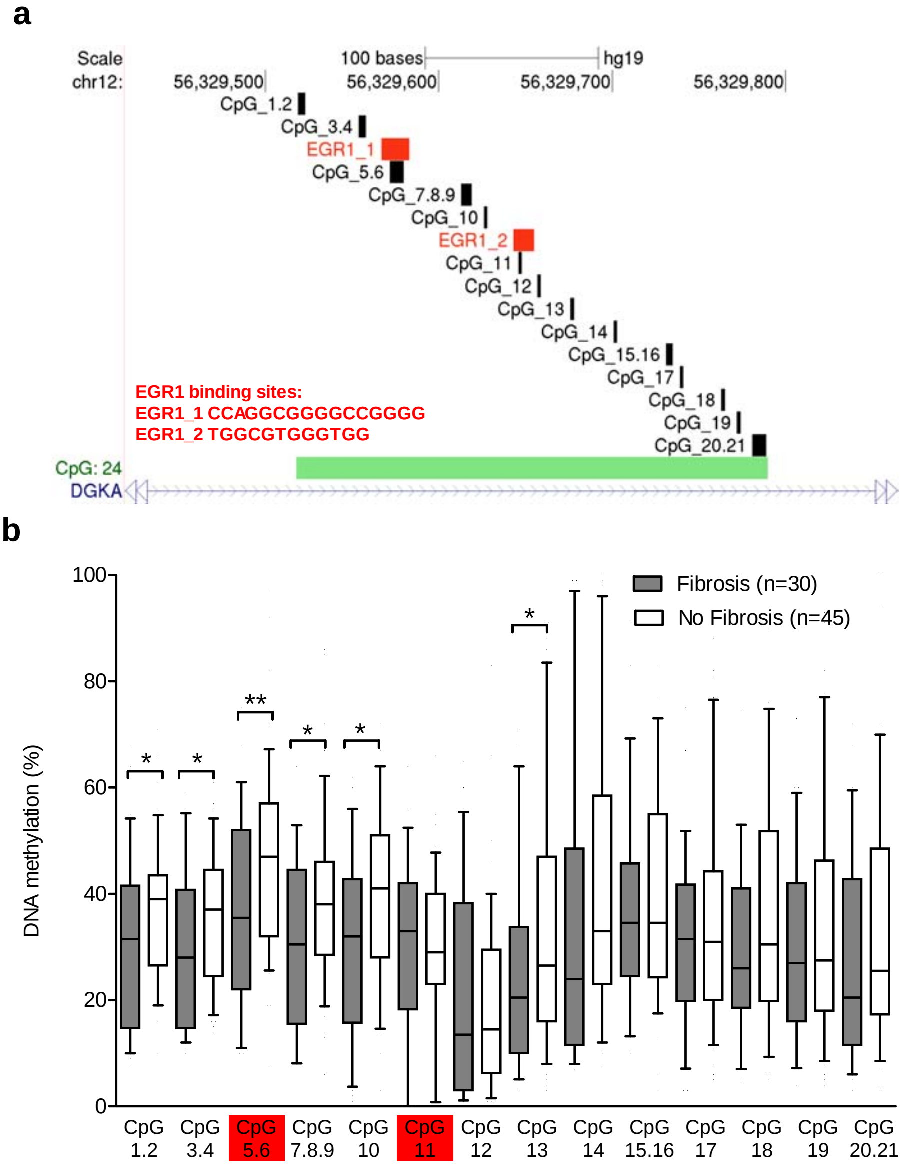

Supplementary Figure 11. High resolution DNA methylation mapping using EpiTYPER assay at the *DGKA* DMR region indicates differential methylation at a conserved EGR1 binding motif. (a) Map of the *DGKA* DMR with *DGKA* transcript (blue), CpG islands (green), single CpGs analyzed with EpiTYPER (black bars) and predicted EGR1 binding sites (red bars). Predicted EGR1 consensus sequences are depicted below in red. (**b**) DNA methylation in fibroblasts derived from patients later developing radiation fibrosis (n=30, grey) and a control cohort (n=45, white). CpG sites overlapping with EGR1 binding motives are marked in red. Graph shows box plots with whiskers (10 - 90% percentile) * $p<0.05$, ** $p<0.01$, Mann-Whitney test.

Supplementary Figure 12

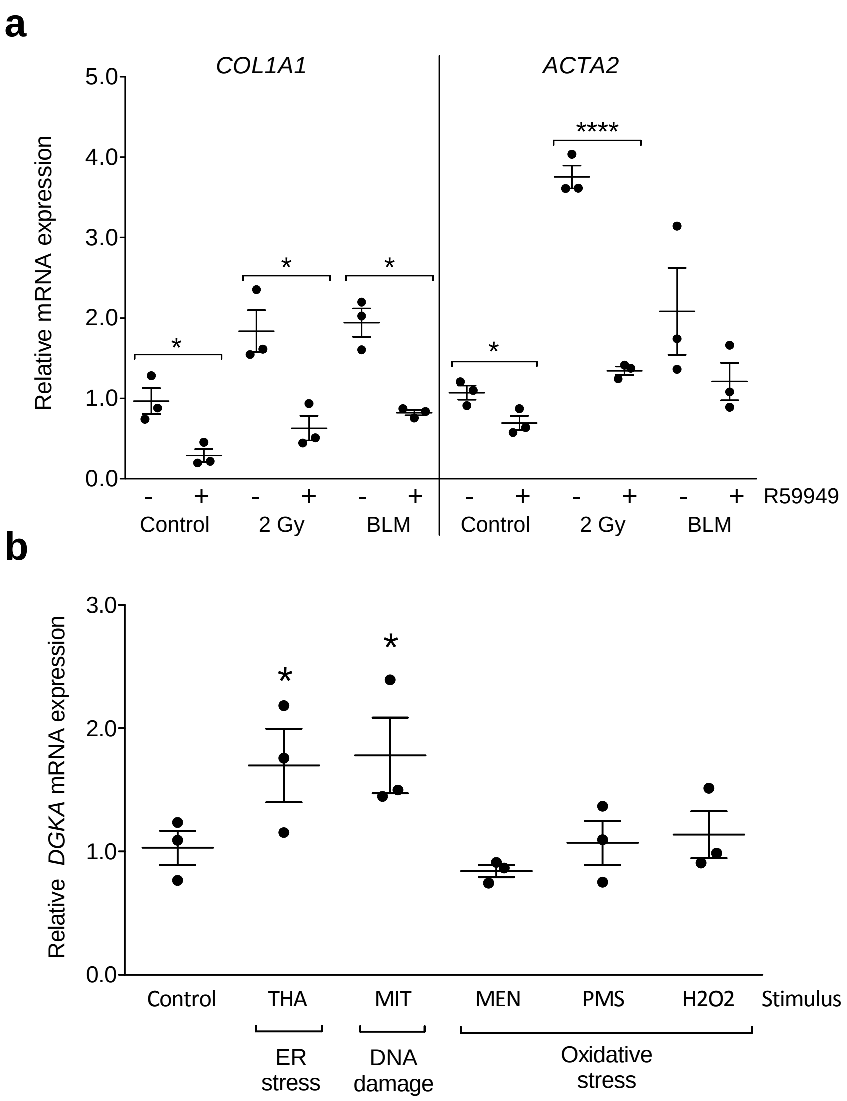

Supplementary Figure 12. DGKA induction is involved in fibroblast activation marker expression after stress exposure. (**a**) Effect of DGKA inhibition using R59949 on the mRNA increase of *COL1A1* and the fibroblast activation marker alpha smooth muscle actin (ACTA2) 48h after radiation (2 Gy) or bleomycin (BLM; 40 µM) treatment. * $p<0.05$, **** $p<0.0001$, Student's t test. (**b**) Stress inducers with different modes of action (endoplasmic reticulum (ER) stress, DNA damage and oxidative stress) affect *DGKA* mRNA expression after 48h exposure. All data depict mean ± SEM of data from duplicate experiments in NHDF (n=3). * $p<0.05$ compared to control treatment, Student's t test.; THA: 0.3 µM thapsigargin; MIT: 1.0 µM mitoxanthrone; MEN: 10.0 µM menadione; PMS: 3.0 µM phenazine methosulfate; H2O2: 250 µM hydrogen peroxide.

Supplementary Figure 13

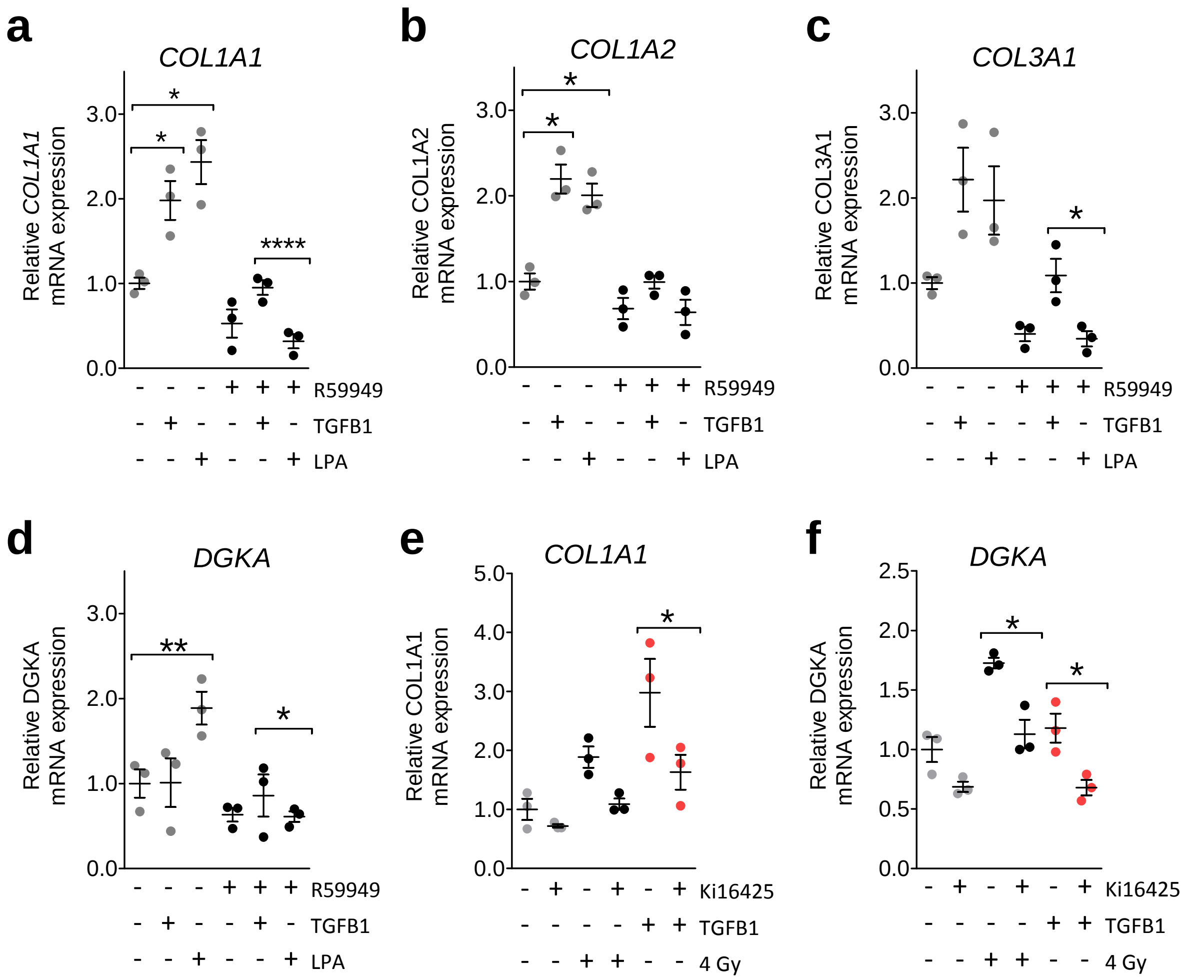

Supplementary Figure 13. Effect of LPA exposure on collagen synthesis and *DGKA* transcriptional induction. *COL1A1* (**a**), *COL1A2* (**b**), *COL3A1* (**c**) and *DGKA* (**d**) mRNA expression was monitored in NHDF treated with R59949 (5.0 µM) for 48h and subsequently exposed to TGFB1 (4 ng ml^{-1}) or LPA (10 µM) during another 48h with inhibitor treatment. (e,f) Effect of the LPA receptor antagonist Ki16425 on *COL1A1* (**e**) and *DGKA* (**f**) expression in NHDF under radiation or TGFB1 exposure. NHDF treated with Ki16425 (5.0 µM) for 48h and then exposed to TGFB1 (4 ng ml^{-1}) or 4 Gy ionizing radiation during another 48h with inhibitor treatment. Data show mean ± SEM of 3 NHDF performed in duplicates. * p<0.05; ** p<0.01, Student's t test.

Supplementary Figure 14

a

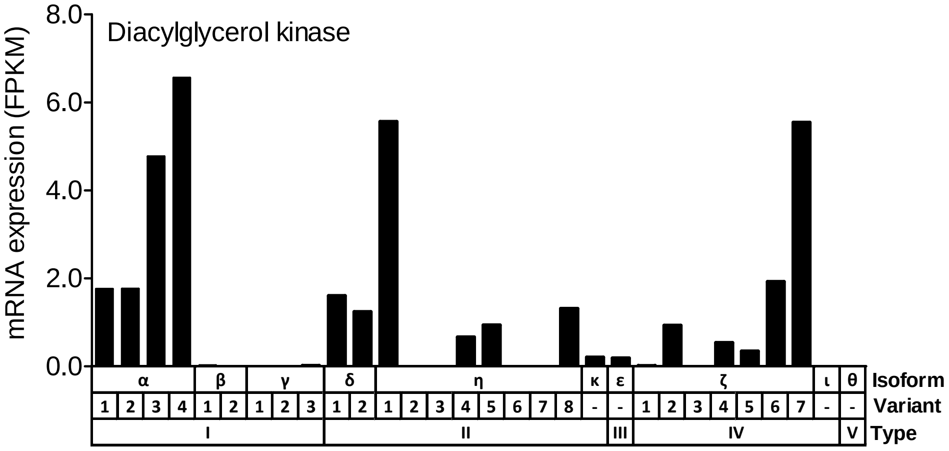

b

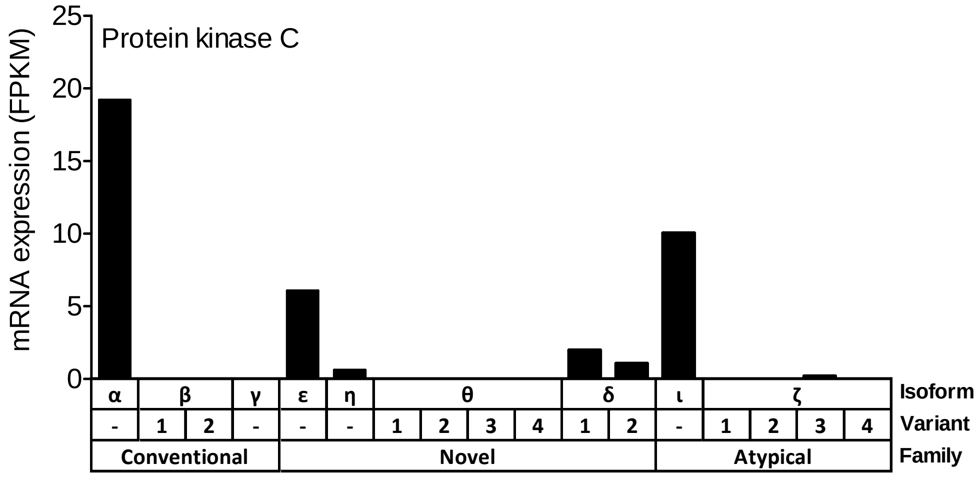

Supplementary Figure 14. mRNA expression patterns of diacylglycerol kinase and PKC isoforms in normal human dermal fibroblasts (NHDF) reveal specific isoform expression patterns. Data show mRNA expression of diacylglycerol kinase isoforms (**a**) and PKC isoforms (**b**) measured by genome-wide mRNA sequencing in primary NHDF derived from a healthy female donor age-matched to the study cohort. Sequence reads were classified according to aligned transcript variant and subtype. FPKM: Fragments per kilobase of transcript per million fragments sequenced.

Supplementary Figure 15

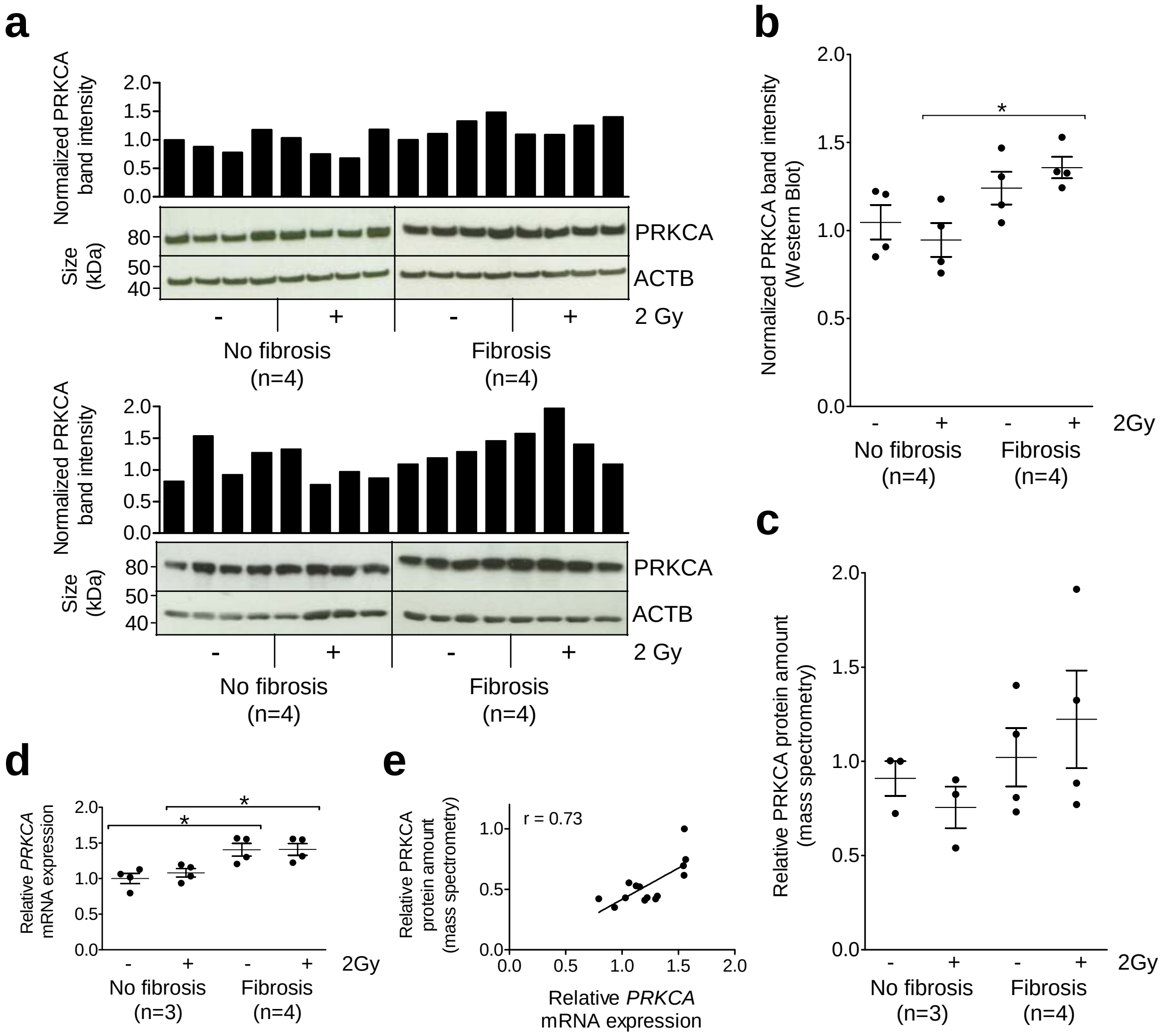

Supplementary Figure 15. PRKCA protein and mRNA expression data in patient fibroblasts show a moderate mRNA expression increase in high risk individuals. (a,b) PRKCA band intensities determined by Western Blot. Images have been cropped to highlight specific bands. Data show two replicate experiments (**a**) and average band intensities for each fibroblast sample normalized to ACTB bands (**b**). (**c**) PRKCA protein levels determined by mass spectrometry in fibroblasts from patients later developing fibrosis (fibrosis) and fibrosis-free patients (No fibrosis). (**d**) *PRKCA* mRNA expression levels in 4 patient fibroblast samples. (**e**) Linear correlation and Pearson correlation coefficient (r) of *PRKCA* mRNA and protein signal derived from mass spectrometry. Data from patient fibroblasts after 48h with and without radiation exposure (n=8 for mRNA expression and Western Blot; n=7 for protein mass spectrometry) are shown. All data show mean±SEM of duplicate experiments. * $p<0.05$, Student's t test.

Supplementary Figure 16

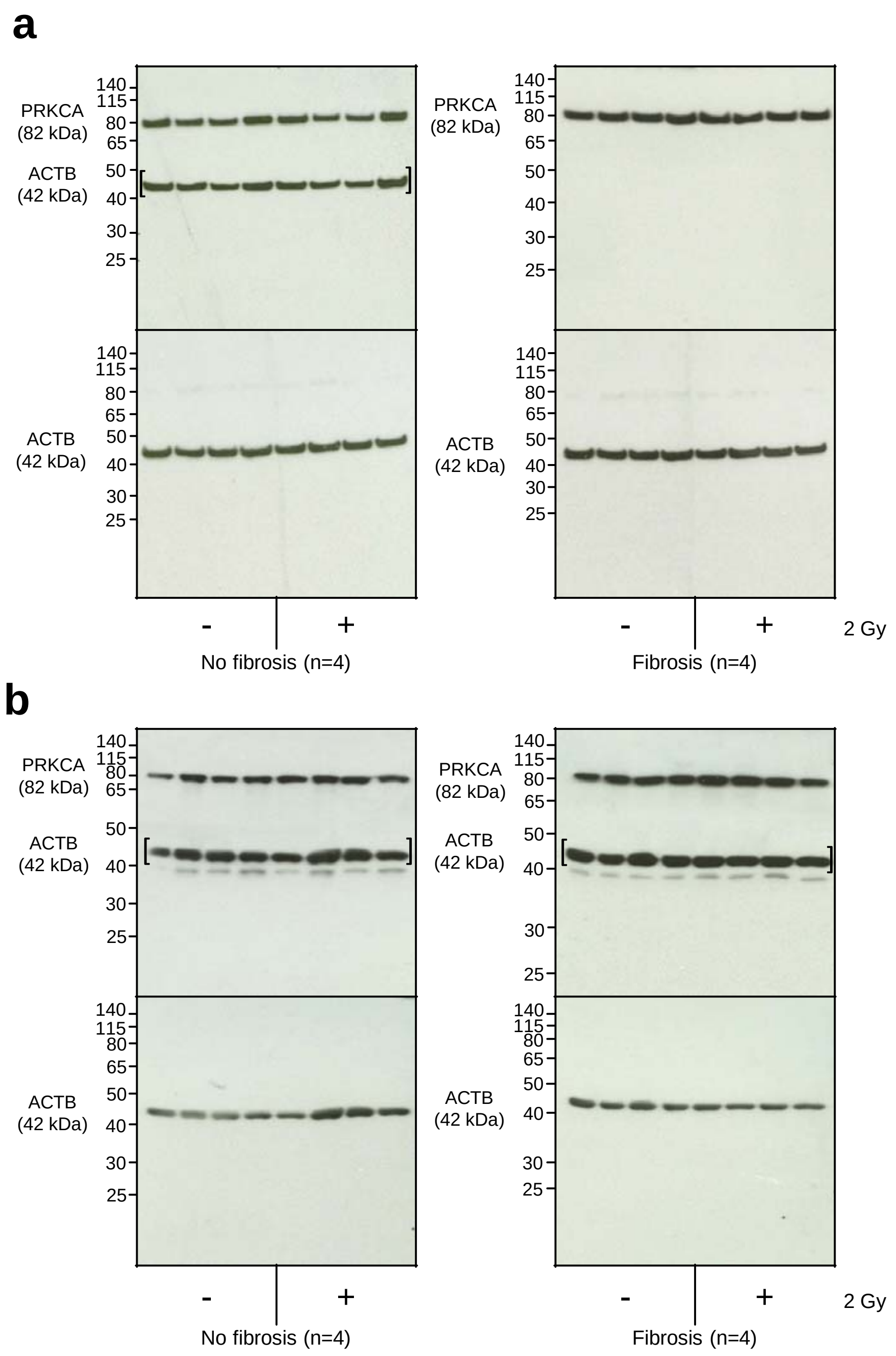

Supplementary Figure 16. Uncropped Western Blot images for PRKCA detection in patient fibroblasts (n=8). Graph shows uncropped images of PRKCA and ACTB-stained Western Blot images from Supplementary Figure 15a. Duplicate experiments (**a,b**) are shown. Proteins and corresponding sizes are indicated. Size ladders (left) show marker size in kDa. Co-stained protein bands (in brackets) were not used for quantitative analysis shown in Supplementary Figure 15b.

Supplementary Figure 17

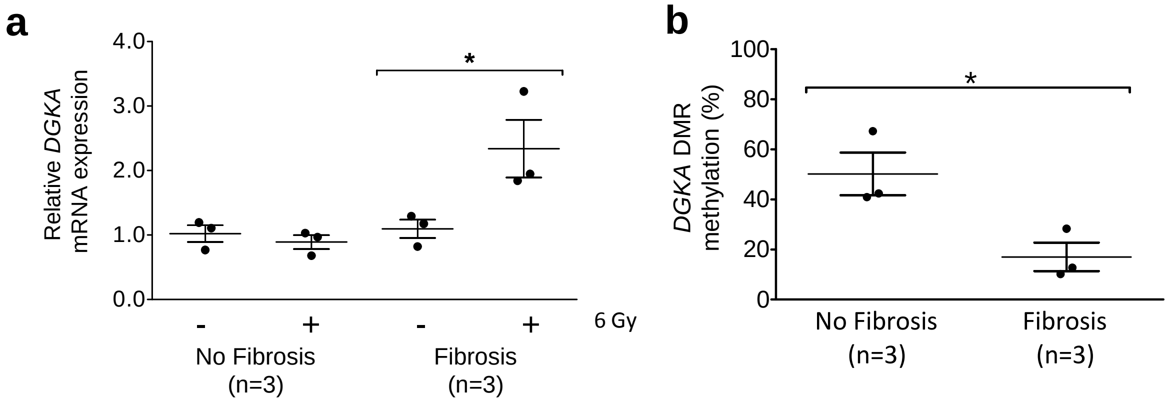

Supplementary Figure 17. Fibroblasts used for drug synergy experiments show different epigenetic regulation of *DGKA*. (**a**) *DGKA* mRNA expression upon radiation exposure (6 Gy) is increased in fibroblasts from fibrosis patients. (**b**) Mean *DGKA* DMR methylation difference of the two fibroblast groups (n=3 each) shows lower methylation in fibrosis patient fibroblasts. Data show mean±SEM from patient fibroblasts (n=3), each determined in duplicate experiments. * $p<0.05$, Students's t test.

Supplementary Figure 18

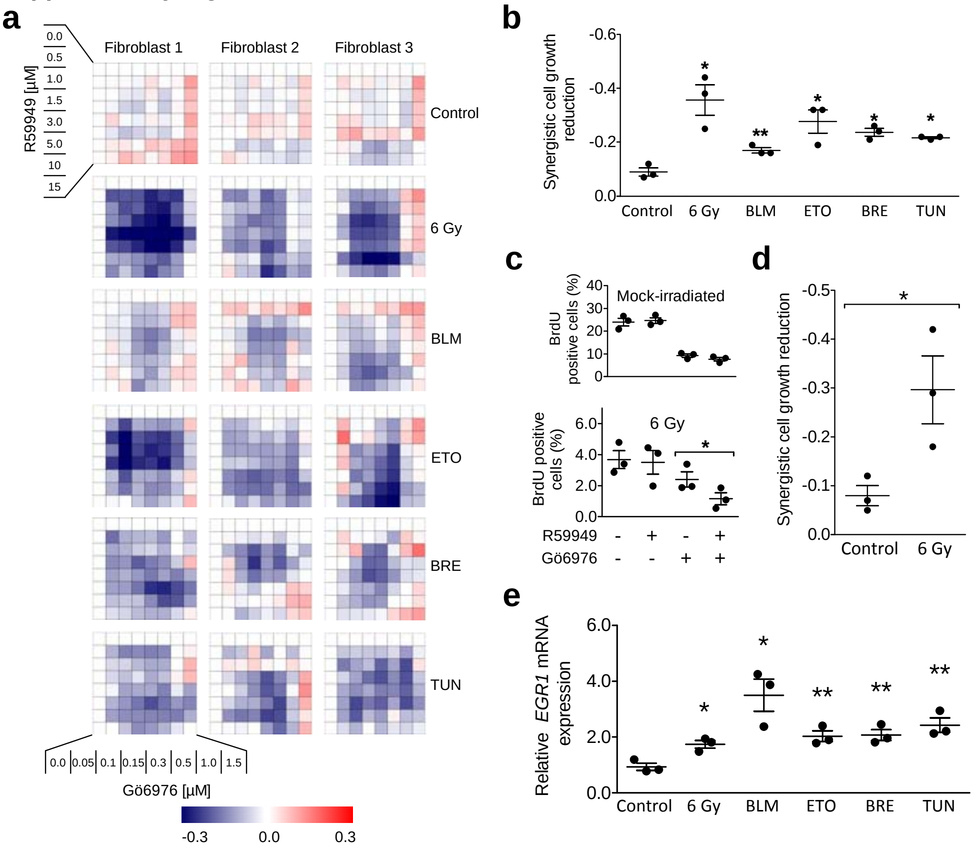

Supplementary Figure 18. Co-inhibition of DGKA and PRKCA has synergistic effects on cell viability of NHDF under stress conditions. (**a**) NHDFs were treated with drug combinations as indicated and co-exposed to a stress stimulus. Relative cell viability (calcein fluorescence assay) difference of the expected (additive effect) compared to the measured value are depicted. Additive or synergistic drug effects were calculated with the Bliss independence model comparing the expected (additive) effects with the observed effects. Scale bar depicts the observed viability differences in comparison to the expected additive effects as blue (synergistic growth suppression) to red (synergistic growth increase). Data depict means of 3 NHDFs measured in quadruplicates. (**b**) Summary of synergistic growth inhibition under combined drug treatment with stress stimuli. Data show most pronounced synergistic growth reduction observed in the dose ranges shown in Supplementary Fig. 18a. (**c**) Confirmation of drug synergy with a bromodeoxyuridine incorporation assay in NHDF after 6 Gy gamma radiation (6 Gy) or mock treatment (mock-irradiated) and co-treatment with R59949 (5.0 µM) and/or Gö6976 (0.5 µM). (**d**) Synergistic growth inhibition in irradiated NHDF under R59949 and Gö6976 co-treatment as determined by bromodeoxyuridine (BrdU) incorporation assay. (**e**) Transcriptional induction of *EGR1* in fibroblasts upon exposure to the selected stress stimuli. Dermal fibroblasts were treated with different stress inducers and mRNA expression was analyzed after 48 h. All data depict mean ± SEM from 3 NHDF determined in duplicate experiments. 6 Gy: 6 Gy gamma irradiation; TUN: 0.75 µM tunicamycin; BLM: 40 µM bleomycin; BRE: 0.1 µM brefeldin A; ETO: 50 µM etoposide. * p<0.05; ** p<0.01 compared to control treatment, Student's t test.

Supplementary Figure 19

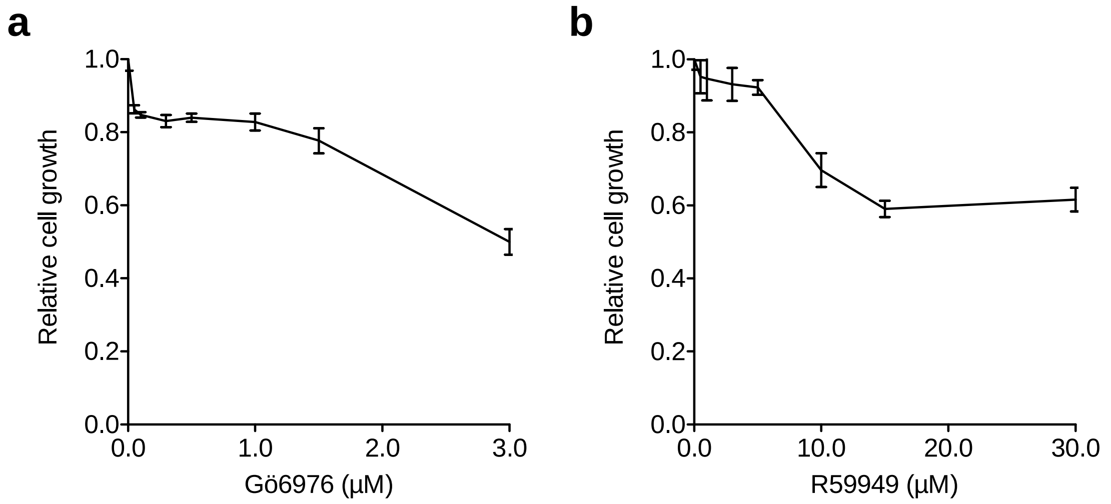

Supplementary Figure 19. DGKA and PRKCA inhibition dose-dependently affects fibroblast cell viability. Cell viability was measured using a calcein fluorescence assay. Data depict mean ± SEM of data from triplicate measurements in NHDF (n=3) exposed to different doses of the PRKCA inhibitor Gö6976 (**a**) or the DGKA inhibitor R59949 (**b**).

Supplementary Figure 20

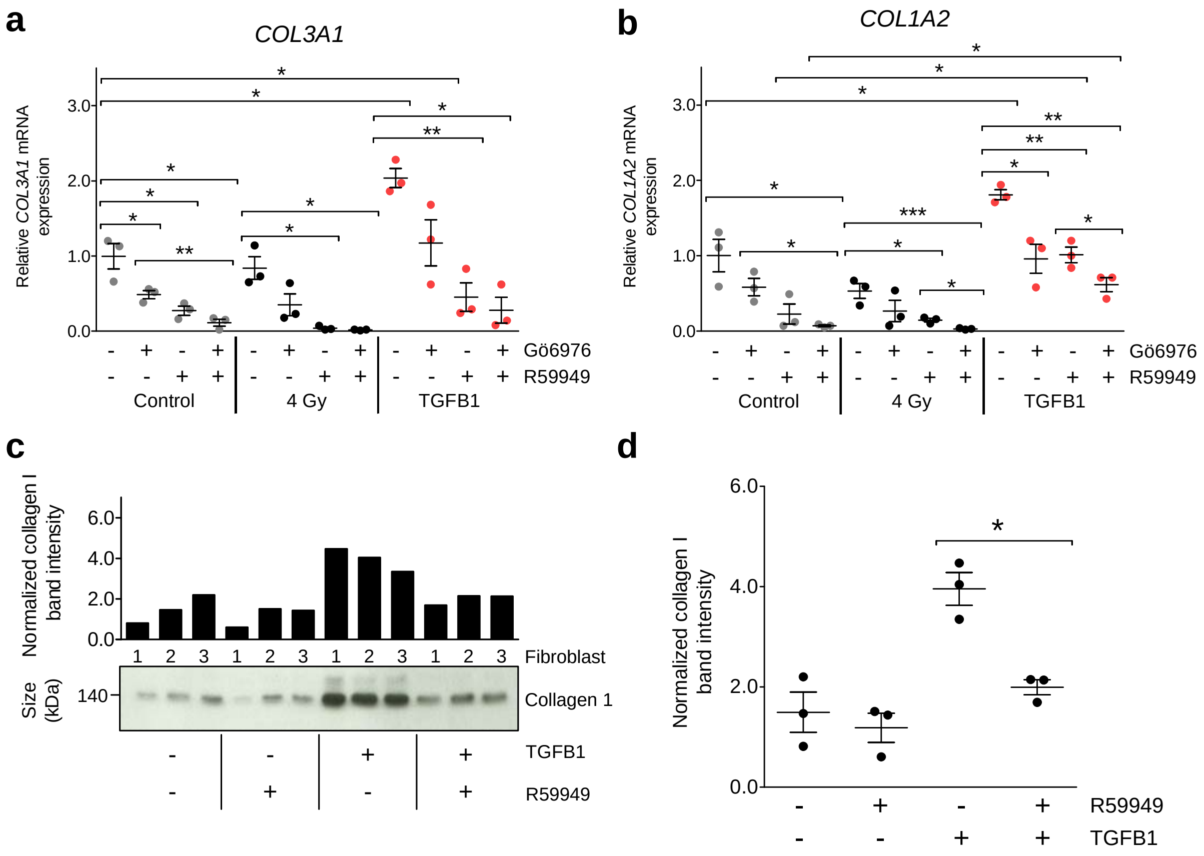

Supplementary Figure 20. PRKCA and DGKA inhibitors suppress collagen expression in NHDF. NHDF were treated with R59949 (5.0 µM), Gö6976 (0.5 µM) or a combination for 48h and then exposed to TGFB1 (4 ng ml^{-1}) or ionizing radiation for another 48h with continued drug treatment. *COL3A1* (**a**) and *COL1A2* (**b**) mRNA expression were assessed. Data show mean ± SEM of 3 NHDF performed in duplicates.* p<0.05; ** p<0.01, *** p<0.001, **** p<0.0001, Student's t test (**c,d**) Collagen 1 determined from total secreted protein in cell culture supernatant of NHDF (n=3) exposed to TGFB1 (4 ng ml^{-1}) and R59949 (5.0 µM) as described above. Data show relative band intensities in Western Blot analysis, with signals normalized to cell number (c), and group-wise comparison with mean±SEM being shown (d). Western Blot images have been cropped to highlight specific bands.

Supplementary Figure 21

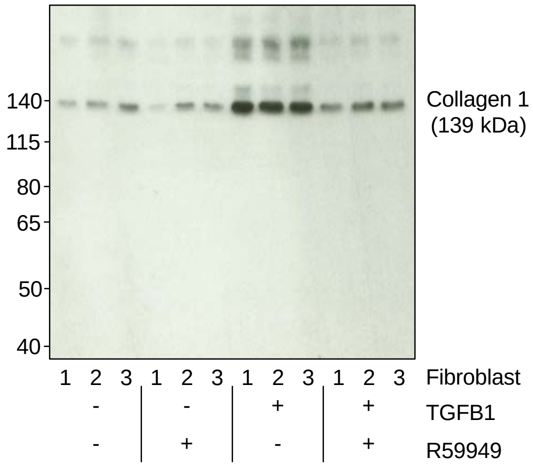

Supplementary Figure 21. Uncropped Western Blot images for collagen 1 detection in NHDF (n=3). Graph shows uncropped images of anti-collagen 1-stained Western Blot images from Supplementary Figure 20c. Proteins and corresponding sizes are indicated. Size ladders (left) show marker size in kDa.

Supplementary Figure 22

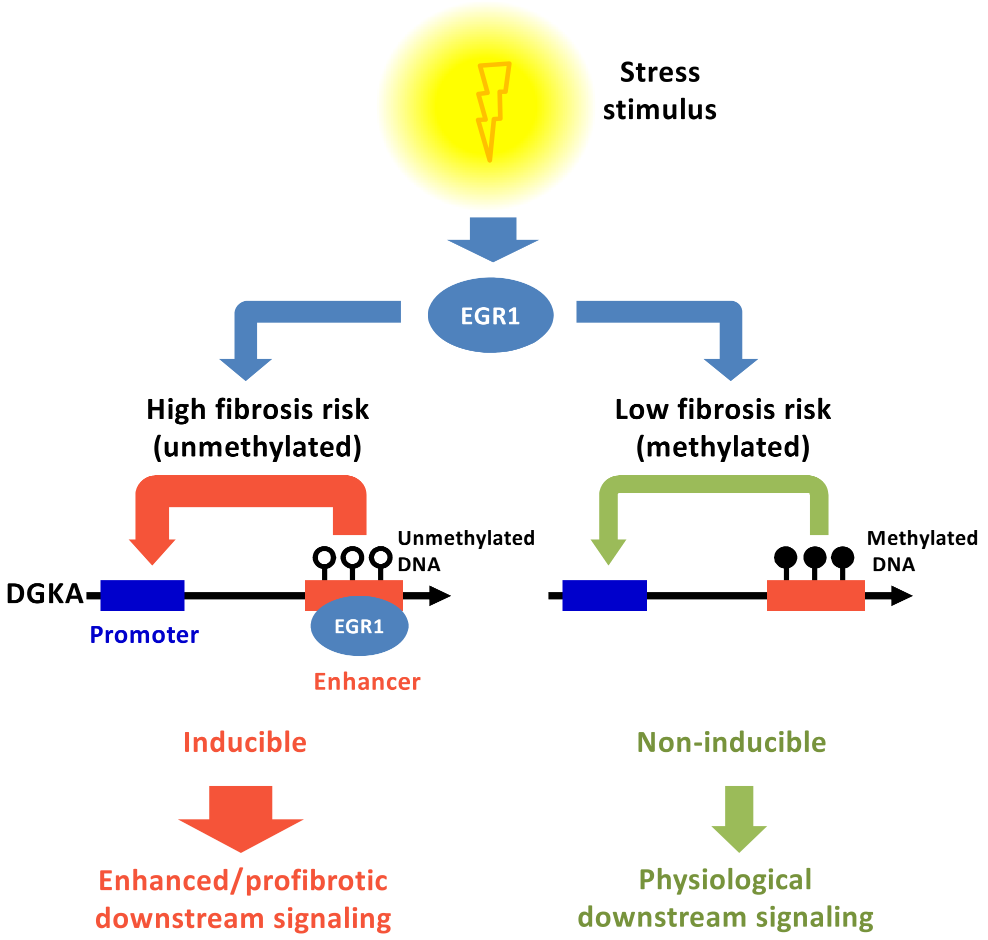

Supplementary Figure 22. Fibrosis risk modulation by DNA methylation at the *DGKA* locus involves EGR1-dependent induction of *DGKA* and exacerbated downstream signaling. Fibrosis risk upon exposure to a stress stimulus such as ionizing radiation is increased by EGR1-mediated inducibility of *DGKA* transcription. Fibroblasts with high DNA methylation at the intragenic DGKA enhancer are protected against stress-induced *DGKA* induction (right branch), while decreased DNA methylation at the enhancer site will allow EGR1 to bind and enhance DGKA downstream profibrotic signaling events (left branch).

Supplementary Table 1. Characteristics of the breast cancer patient cohort used for genome-wide DNA methylation analysis.

	Samples analyzed on 450K methylation arrays		Samples used in MassArray analysis	
**Characteristics**	**No fibrosis (n=12)**	**Fibrosis (n=12)**	**No fibrosis (n=45)**	**Fibrosis (n=30)**
Age at intraoperative radiation (years, median (min-max))	65.7 (47.2-74.1)	63.1 (42.3-72.1)	61.2 (35.1-74.1)	60.7 (31.5-72.2)
Intraoperative radiation dose (Gy, median (min-max))	20 (6-20)	20 (20-20)	20 (6-20)	20 (20-20)
External beam radiation dose[a] (Gy, median (min-max))	46 (46-50)	46 (46-46)	46 (46-50)	46 (46-50)
Age at fibrosis (years, median (min-max))		64.3 (43-73)		62.7 (34-78)
Follow-up time until year 5 after IORT (years, median (min-max))	5.0 (4.3-5.2)	5.0 (3.1-5.3)	4.7 (2.0-5.4)	4.9[b] (2.2-5.5)
Body mass index (kg m^{-2}, median (min-max))	27.3 (22-42)	26.9 (22-38)	25.6 (18-42)	27.5 (21-38)
Smoker[c]	3 25%	6 50%	13 20%	10 27%
Chemotherapy prior to fibroblast sampling[d]	3 25%	2 17%	16 36%	8 27%
Tumor size: T1	8 67%	5 42%	28 62%	18 60%
T2	4 33%	7 58%	17 38%	12 40%
Nodal status: N0	6 50%	10 83%	31 69%	23 77%
N1	6 50%	2 17%	11 24%	6 20%
N2			3 7%	
N3				1 3%
Estrogen receptor score >2	10 83%	8 68%	28 62%	19 63%
Histological grading: grade 1	3 25%	1 8%	8 18%	2 7%
grade 2	6 50%	9 75%	22 49%	20 67%
grade 3	3 25%	2 17%	4 9%	5 17%

[a]External beam radiation was given at doses of 2 Gy per fraction.[b] For one patient, fibrosis was observed at follow-ups after 1 and 6 year. As the patient had no observation in between these time points, she was not counted in median and range. [c] Smoking status refers to tobacco smoking (ever-smoker: yes/no). [d] Chemotherapy refers to any treatment regimen including the use of epirubicine, 5-fluorouracil, cyclophosphamide, docetaxel or paclitaxel. Chemotherapy data are missing for 6 patients of the extended sample set.

Supplementary Table 2. Differentially methylated CpG dinucleotides in patient-derived skin fibroblasts associated with fibrosis onset.

Ilumina probe ID	Adjusted p-value	Gene name	Probe localization relative to gene	Mean methylation beta value difference (control versus case)[1]
cg27295963	0.01023	MT3	TSS1500	0.35
cg01224520	0.01156	n.d.	Intergenic	0.34
cg21468035	0.01433	ABHD1	TSS1500	0.24
cg01310482	0.00152	CUX1	Body	0.24
cg02052797	0.01582	n.d.	Intergenic	0.24
cg07029024	0.00355	n.d.	Intergenic	0.24
cg16613029	0.04223	USP7	Body	0.22
cg14215105	0.00743	INTS3	Body	0.22
cg03630771	0.00013	n.d.	Intergenic	0.22
cg01734062	0.00617	TMEM92	1stExon	0.22
cg26272088	0.03982	IGF1R	Body	0.22
cg06739462	0.01447	DGKA	5'UTR	0.22
cg06915826	0.03237	DGKA	5'UTR	0.21
cg09801924	0.03167	RELA	Body	0.21
cg11918822	0.03331	VRK3	3'UTR	0.21
cg06651376	0.04466	SMYD4	Body	0.21
cg15822411	0.00097	CCDC83	5'UTR	0.17
cg24726783	9.535E-05	SVIL	Body	0.17
cg22942576	2.813E-05	CSRP1	TSS1500	0.16
cg04960065	2.122E-10	n.d.	Intergenic	0.15
cg26831562	0.00104	THRAP3	3'UTR	0.14
cg07215975	0.00109	PTPRE	5'UTR	0.12
cg16380876	3.078E-05	PTPRE	5'UTR	0.04
cg08780106	0.00948	SVIL	Body	0.11
cg10413352	2.581E-06	n.d.	Intergenic	-0.18
cg18075011	0.01433	STAB1	Body	-0.21
cg04569190	0.01731	CDC42BPB	Body	-0.21
cg15154047	0.03237	n.d.	Intergenic	-0.21
cg07147475	0.03666	n.d.	Intergenic	-0.22
cg08383160	0.00925	n.d.	Intergenic	-0.23
cg23689428	0.01717	LOC84931	TSS200	-0.23
cg09015774	0.00021	CAMTA1	Body	-0.24
cg15432303	0.02987	n.d.	Intergenic	-0.25
cg14323117	0.03204	n.d.	Intergenic	-0.26
cg14711869	0.01731	FAM193A	Body	-0.28

n.d.: not defined, UTR: Untranslated region, TSS200/1500: 200 bp/1500 bp window around transcription start site, Body: Gene body/intragenic localization, 1stExon: Localization within the first exon of a known transcript,Intergenic: No genomic context assigned.

Supplementary Table 3. Transcription factor binding site (TFBS) prediction at the *DGKA* DMR using different algorithms for binding site identification.

PROMO [7]	ConSite [8]	TRANSFAC [9]	JASPAR [10]
STAT4	AML-1	KID3	MZF1_1-4
TFII-I	Thing1-E47	PAX4	ZNF354C
AP-2alphaA	E2F	MYOGNF1	PDR1
C/EBPbeta	RREB-1	AP4	PDR3
GR-alpha	Hen-1	AHR	HAL9
ENKTF-1	RORalfa-2	EGR1	Arnt::Ahr
GR-beta	SOX17	P300	RDS2
c-Jun	RORalfa-1	PAX5	CRZ1
c-Myb	NRF-2	ZF5	NFE2L1::MafG
RXR-alpha	CREB	MYB	SKN7
Pax-5	c-FOS		ARG80
p53	SAP-1		ARG81
c-Ets-1	c-REL		ASG1
Elk-1			CAT8
YY1			
XBP-1			
ER-alpha			
E2F-1			
EGR1			
SP3			

Supplementary Table 4. UPLC-gradient used for the elution of (lyso)phophatidic acids ((L)PAs) for UPLC-ESI-MS/MS in NHDF.

Time (min)	Flow rate (ml min^{-1})	Solvent A (%)	Solvent B (%)	Curve
Initial	0.35	70	30	Initial
0.20	0.35	70	30	Linear
0.40	0.35	65	35	Linear
5.00	0.35	5	95	Linear
7.00	0.35	5	95	Linear
7.50	0.35	70	30	Linear
8.50	0.35	70	30	Linear

Supplementary Table 5. Diacylglycerols (DAGs) analyzed with UPLC-ESI-MS/MS in NHDF.

DAG	DAG (side chains)	R_t (min)	M_W [u]	m z^{-1} $[DAG+NH_4]^+$	m z^{-1} $[MAG1 +H]^+$	m z^{-} $^{1}[MAG2+H]^+$
**DAG 30:0**	DAG (14:0/16:0)	1.86	540.5	558.5	285.2	313.2
**DAG 32:0**	DAG (16:0/16:0)	2.29	568.5	586.5	313.3	-
**DAG 32:2**	DAG (14:0/18:2)	1.64	564.5	582.5	285.2	337.3
**DAG 34:0**	DAG (16:0/18:0)	2.74	596.5	614.6	313.3	341.3
**DAG 34:1**	DAG (16:0/18:1)	2.35	594.5	612.6	339.3	313.3
**DAG 34:2**	DAG (16:0/18:2)	2.03	592.5	610.5	313.3	337.3
**DAG 34:3**	DAG (16:0/18:3)	1.79	590.5	608.5	313.3	335.3
**DAG 36:1**	DAG (18:0/18:1)	2.79	622.6	640.6	339.3	341.3
**DAG 36:2**	DAG (18:1/18:1)	2.42	620.5	638.6	339.3	-
**DAG 36:4**	DAG (16:0/20:4)	1.96	616.5	634.5	313.3	361.3
**DAG 38:4**	DAG (20:4/18:0)	2.41	644.5	662.6	361.3	341.3
**DAG 38:5**	DAG (20:5/18:0)	2.13	642.5	660.6	341.3	359.3
**DAG 38:6**	DAG (22:6/16:0)	1.88	640.5	658.5	385.3	313.3
**IS**	DAG (17:0/17:0)-d5	2.69	601.6	619.6	332.3	-

R_t: Retention time, MW [u]: Molecular weight of DAG or corresponding monoacylglycerol (MAG). m z^{-1}: Mass-to-charge ratio of the target ammoniated DAG and protonated MAG ions; IS: Internal standard.

Supplementary Table 6. (Lyso)phosphatidic acids ((L)PAs) analyzed with UPLC-ESI-MS/MS in NHDF.

Lipid	Rt (min)	m z^{-1}
LPA (16:1)	1.37	407.3
LPA (16:0)	1.62	409.2
LPA (18:2)	1.49	433.3
LPA (18:1)	1.71	435.3
LPA (18:0)	2.03	437.2
LPA (20:4)	1.47	457.3
PA (34:2)	3.59	671.50
PA (34:1)	3.77	673.40
PA (36:4)	3.59	695.50
PA (36:2)	3.80	699.40
PA (36:1)	3.98	701.50
IS	3.72	704.60
PA (38:5)	3.65	721.50
PA (38:4)	3.82	723.50
PA (38:2)	4.00	727.50

Rt: Retention time, m z^{-1}: Mass-to-charge ratio,
IS: Internal standard.

Supplementary Table 7. Candidate proteins reported to be associated with DGKA.

Refseq name	Uniprot name	Protein name	Reference
RELA	TF65_HUMAN	v-rel avian reticuloendotheliosis viral oncogene homolog A	[11]
MMP9	MMP9_HUMAN	Matrix metallopeptidase 9	[12]
ITGB1	ITB1_HUMAN	Integrin, beta 1	[12]
ARHGDIA	GDIR_HUMAN	Rho GDP dissociation inhibitor (GDI) alpha	[13]
RAC1	RAC1_HUMAN	Ras-related C3 botulinum toxin substrate 1 (rho family, small GTP binding protein Rac1)	[13]
RASGRP1	GRP1_HUMAN	RAS guanyl releasing protein 1	[14-17]
MAPK3	MK03_HUMAN	Mitogen-activated protein kinase 3 (= ERK1)	[14-18]
MAPK1	MK01_HUMAN	Mitogen-activated protein kinase 1 (= ERK2)	[14-18]
MAPK8	MK08_HUMAN	Mitogen-activated protein kinase 8 (= JNK1)	[15,18]
MAPK9	MK09_HUMAN	Mitogen-activated protein kinase 9 (= JNK2)	[15,18]
FOS	FOS_HUMAN	FBJ murine osteosarcoma viral oncogene homolog	[19]
RAF1	RAF1_HUMAN	v-raf-1 murine leukemia viral oncogene homolog 1	[19]
CCND3	CCND3_HUMAN	Cyclin D3	[19]
MTOR	MTOR_HUMAN	Mechanistic target of rapamycin (serine/threonine kinase)	[20]
PRKCZ	KPCZ_HUMAN	Protein kinase C, zeta	[11-13]
PRKCI	KPCI_HUMAN	Protein kinase C, iota	[12]
PRKCE	KPCE_HUMAN	Protein kinase C, epsilon	[a]
PRKCH	KPCL_HUMAN	Protein kinase C, eta	[a]
PRKCD	KPCD_HUMAN	Protein kinase C, delta	[a]
PRKCA	KPCA_HUMAN	Protein kinase C, alpha	[a]

[a]Protein kinase C isoforms were included based on evident mRNA expression in primary dermal fibroblasts (RNA-seq data).

Supplementary Table 8. DNA primers used for DNA methylation, cloning and quantitative real-time PCR analysis.

Name (UPL hydrolysis probe)	Forward primer	Reverse primer
EpiTYPER primers for methylation analysis[a]		
DGKA_DMR_1	GATTGGGAAATATTAGATTTGTTGG	TTCCTAACCATAACCCCATTTTATT
DGKA_DMR_2	AATAAAATGGGGTTATGGTTAGGA	ACCTTCCCTAACAATATCTATCCTC
DGKA_DMR_3	AAGAATGGGATTTAGATATGATTGA	AATACCCACAAAATCTCTTCCTCA
DGKA_DMR_4	GATTGGGAAATATTAGATTTGTTGG	AACTCTAACAAAATCCCTAACCCC
DGKA_5'UTR_1	GAGTAGGGGAGAGTTTGGTATGTTT	AAATCCCATTCTTAACTCTAATACCAC
DGKA_5'UTR_2	TAGATGTGTTTATGATTGGAAATGG	CAACTACCACTCACATCTCCTTAAA
DGKA_5'UTR_3	TTAGGTTGGTTTAGGAGTTAAAGGG	ACCTCACTTCAAAAAAACAACTCAC
DGKA_5'UTR_4	AAGAGGGATATAGGGAAAGGAAGAT	AAAACTAAAACAAACAATCCAAATATAAC
DGKA_5'UTR_5	AAGAGGGATATAGGGAAAGGAAGAT	CCTTACACAATACAACCCAATTAAA
DGKA_5'UTR_6	GTTTATTGTTATTTTGATTATTGTTTTAGG	ACTCCTAAACCAACCTAACTATTCTCC
DGKA_5'UTR_7	GATAGAATGGAATAGATAGAAGAAAAGAT	CCTTACACAATACAACCCAATTAAA
DGKA_5'UTR_8	GAGATATTTTAGTGTTTATTGTTATTTTGA	ACTCCTAAACCAACCTAACTATTCTCC
DGKA_5'UTR_9	GAGATATTTTAGTGTTTATTGTTATTTTGA	CCTTTAACTCCTAAACCAACCTAACT
DGKA_PROMOTER_1	AGTGAGTAGGATGTTTGGGTTTTTA	CACCACTAACTTCACACTCAAAAAAT
DGKA_PROMOTER_2	TAGGTTATGGTGAATTGGAAATTTAG	CTAAAAACCAAACAAAAACCCTTCT
LINE1	TTTATATTTTGGTATGATTTTGTAG	TTTATCACCACCAAACCTACCCT
RT-qPCR primers		
DGKA (#52)	GATCTGGCTCGATGCCTAAG	GATCTTTGCCAGATTCTGTCCT
ACTA2 (#35)	AGCCAAGCACTGTCAGGAAT	TTGTCACACACCAAGGCAGT
COL1A1 (#67)	GGGATTCCCTGGACCTAAAG	GGAACACCTCGCTCTCCA
COL1A2 (#79)	CTGGAGAGGCTGGTACTGCT	AGCACCAAGAAGACCCTGAG
COL3A1 (#20)	CTGGACCCCAGGGTCTTC	GACCATCTGATCCAGGGTTTC
EGR1 (#54)	AGCCCTACGAGCACCTGAC	GGGCAGTCGAGTGGTTTG
ACTB (#11)	ATTGGCAATGAGCGGTTC	GGATGCCACAGGACTCCAT
GAPDH (#60)	GCCCAATACGACCAAATCC	AGCCACATCGCTCAGACAC
HPRT1 (#73)	TGACCTTGATTTATTTTGCATACC	CGAGCAAGACGTTCAGTCCT
Primers for cloning and mutagenesis of DGKA sequences[b]		
**DGKA_DMR**	**TACCCAGAGTCTCTTCCCCT**	**TTTCTGCGCTTTCTTCCACC**
DGKA_1	TGTTTCCCCTACAGCCTGAG	GCAGGGCTGAAGTACAATCG
DGKA_2	CAATGGCTGACTAGGACCTTT	GACACTCCCCATCCTGTCTT
DGKA_3	CCCTTGCTCTCTTTTCACCG	GACACTCCCCATCCTGTCTT
DGKA_4	ACGCCTCCTTCTTAGATGTTTC	CCAACCCCTGTCTAACTCCC
DGKA_5	CAGCAGGTAAAGTGGGAGGA	GTTCCTGACGACATAGCTGC
DGKA_6	TAAAGTGGGAGGATGAGGGC	AGGCCAAACAAGAACCCTTC
DGKA_7	TTCGAAGTTCCCAGAGTCGG	TGAAGCCCAGTTGTACGTCT
DGKA_8	CCACCTGTCACTGGGAAGTT	TGAAGCCCAGTTGTACGTCT
DGKA_9	TCTGAGTGTTCCCAGAGAGC	GAAGAGGCAGGGGAAGAGAC
DGKA_10	AATCAAGGAAAGTCGCCCAC	CAGAGCGTACAGTGGGAAGA
DGKA_11	GTGGAAGAAAGCGCAGAAAC	GAGTCTGCAAGGTCAGAGCT

DGKA_P1	CAATGGCTGACTAGGACCTTT	GCAGGGCTGAAGTACAATCG
DGKA_P2	CTCGTGTCACCCAGGCTG	CCAACCCCTGTCTAACTCCC
DGKA_P3	TTCGAAGTTCCCAGAGTCGG	AGGCCAAACAAGAACCCTTC
DGKA_ORF	CACCATGGCCAAGGAGAGG	GTACAAGAAAGCTGGGTCCT
DGKA_EGR1_1_mutagenesis	GCAGTCCAGACTAGGCCGGGGCTAG	CTAGCCCCGGCCTAGTCTGGACTGC
DGKA_EGR1_2_mutagenesis	CGTTGCGTGGCACTATTGGCTCGG	CCGAGCCAATAGTGCCACGCAACG
Primers for ChIP-qPCR		
DGKA_ChIP_DMR (#83)	AGAAGCGCTAGAGGTCGTTG	GCCATGACCCCATTTTGT
DGKA_ChIP_promoter (#55)	CCTCCAGGTCCCCAACTT	TTTCAGAAACTTCCCAGTGACA
DGKA_ChIP_control (#2)	CCTGCTTCCACAGTCACATC	GGGGTCCTTATAAGTGAAAGAGG
MMP9 promoter (#53)	GAACCAATCTCACCGACAGG	ACAGCCCTCCCCAACTCTA
PTEN promoter (#23)	CAGGGAGGGGGTCTGAGT	CCGTGTTGGAGGCAGTAGA
TGFB1 promoter (#82)	TTAATCCGGGGGATGAGAC	TGACTCTCCTTCCGTTCTGG
MMP1 promoter (#33)	CTTCCCAGCCTCTTGCTG	CTGGAAGGGCAAGGACTCTAT
Primers for chromatin conformation capture		
DGKA_3C_1 (#34)	AGGTGGAGGTTGCAGTGAG	GGAAGGGTGGGGAACTAGG
DGKA_3C_2 (#34)	AGGGACAGAAAACTGATAGGCA	GGAAGGGTGGGGAACTAGG
DGKA_3C_3 (#34)	CCCAGGTGTAAGAAGGACGAT	GGAAGGGTGGGGAACTAGG
DGKA_3C_4 (#34)	AGGAGCACTCGTTTCCAAGA	GGAAGGGTGGGGAACTAGG
DGKA_3C_5 (#34)	ACAACTGGGCTTCACTGATG	GGAAGGGTGGGGAACTAGG
DGKA_3C_6 (#81)	CTTGTCTGGCAGGGTCTGAG	GTGGGAGTAGAGACAGAATGGA
DGKA_3C_7 (#67)	GTTGCAGTGAGCTGAGATGG	GTGGGAGTAGAGACAGAATGGA
DGKA_3C_input_control (#7)	AAGGTGAGGGTGGTCAGATG	CATCTCTGTCTCTCCATTTCTGG
DGKA_3C_genomic_control (#76)	AGCCACCTTCCAAACATTGC	CCAAAAAGAGTCCAGGACCA

[a]All EpiTYPER primers carry 5' tags: AGGAAGAGA (forward) and CAGTAATACGACTCACTATAGGGAGAAGGCT (reverse), [b]cloning was carried out using XhoI/NheI (pGl4.10) or BamHI/SpeI (pCpG-free-promoter-Lucia)

Supplementary references

1 Consortium, E. P. An integrated encyclopedia of DNA elements in the human genome. *Nature* **489**, 57-74 (2012).

2 Tanjore, H., Lawson, W. E. & Blackwell, T. S. Endoplasmic reticulum stress as a pro-fibrotic stimulus. *Biochim. Biophys. Acta* **1832**, 940-947 (2013).

3 Shin, S. Y., Kim, J. H., Baker, A., Lim, Y. & Lee, Y. H. Transcription factor Egr-1 is essential for maximal matrix metalloproteinase-9 transcription by tumor necrosis factor alpha. *Mol. Cancer Res.* **8**, 507-519 (2010).

4 Virolle, T. *et al.* The Egr-1 transcription factor directly activates PTEN during irradiation-induced signalling. *Nat. Cell Biol.* **3**, 1124-1128 (2001).

5 Presser, L. D., McRae, S. & Waris, G. Activation of TGF-beta1 promoter by hepatitis C virus-induced AP-1 and Sp1: role of TGF-beta1 in hepatic stellate cell activation and invasion. *PLoS ONE* **8**, e56367 (2013).

6 Angel, P. *et al.* Phorbol ester-inducible genes contain a common cis element recognized by a TPA-modulated trans-acting factor. *Cell* **49**, 729-739 (1987).

7 Messeguer, X. *et al.* PROMO: detection of known transcription regulatory elements using species-tailored searches. *Bioinformatics* **18**, 333-334 (2002).

8 Sandelin, A., Wasserman, W. W. & Lenhard, B. ConSite: web-based prediction of regulatory elements using cross-species comparison. *Nucleic Acids Res.* **32**, W249-252 (2004).

9 Wingender, E., Dietze, P., Karas, H. & Knuppel, R. TRANSFAC: a database on transcription factors and their DNA binding sites. *Nucleic Acids Res.* **24**, 238-241 (1996).

10 Sandelin, A., Alkema, W., Engstrom, P., Wasserman, W. W. & Lenhard, B. JASPAR: an open-access database for eukaryotic transcription factor binding profiles. *Nucleic Acids Res.* **32**, D91-94 (2004).

11 Kai, M. *et al.* Diacylglycerol kinase alpha enhances protein kinase Czeta-dependent phosphorylation at Ser311 of p65/RelA subunit of nuclear factor-kappaB. *FEBS Lett.* **583**, 3265-3268 (2009).

12 Rainero, E. *et al.* The diacylglycerol kinase alpha/atypical PKC/beta1 integrin pathway in SDF-1alpha mammary carcinoma invasiveness. *PLoS ONE* **9**, e97144 (2014).

13 Chianale, F. *et al.* Diacylglycerol kinase alpha mediates HGF-induced Rac activation and membrane ruffling by regulating atypical PKC and RhoGDI. *Proc. Natl. Acad. Sci. U. S. A.* **107**, 4182-4187 (2010).

14 Jones, D. R., Sanjuan, M. A., Stone, J. C. & Merida, I. Expression of a catalytically inactive form of diacylglycerol kinase alpha induces sustained signaling through RasGRP. *FASEB J.* **16**, 595-597 (2002).

15 Zha, Y. *et al.* T cell anergy is reversed by active Ras and is regulated by diacylglycerol kinase-alpha. *Nat. Immunol.* **7**, 1166-1173 (2006).

16 Guo, R. *et al.* Synergistic control of T cell development and tumor suppression by diacylglycerol kinase alpha and zeta. *Proc. Natl. Acad. Sci. U. S. A.* **105**, 11909-11914 (2008).

17 Olenchock, B. A. *et al.* Disruption of diacylglycerol metabolism impairs the induction of T cell anergy. *Nat. Immunol.* **7**, 1174-1181 (2006).

18 Prinz, P. U. *et al.* NK-cell dysfunction in human renal carcinoma reveals diacylglycerol kinase as key regulator and target for therapeutic intervention. *Int. J. Cancer* **135**, 1832-1841 (2014).

19 Flores, I. *et al.* Diacylglycerol kinase inhibition prevents IL-2-induced G1 to S transition through a phosphatidylinositol-3 kinase-independent mechanism. *J. Immunol.* **163**, 708-714 (1999).

20 Dominguez, C. L. *et al.* Diacylglycerol kinase alpha is a critical signaling node and novel therapeutic target in glioblastoma and other cancers. *Cancer Discov* **3**, 782-797 (2013).
